# A circuit supporting concentration-invariant odor perception in *Drosophila*

**DOI:** 10.1186/jbiol108

**Published:** 2009-01-26

**Authors:** Kenta Asahina, Matthieu Louis, Silvia Piccinotti, Leslie B Vosshall

**Affiliations:** 1 Laboratory of Neurogenetics and Behavior, The Rockefeller University, 1230 York Avenue, New York, NY 10065, USA; 2 Howard Hughes Medical Institute, The Rockefeller University, 1230 York Avenue, New York, NY 10065, USA; 3 Current address: Howard Hughes Medical Institute, Division of Biology, California Institute of Technology, Pasadena, CA 91125, USA; 4 Current address: EMBL-CRG Systems Biology Unit, Centre for Genomic Regulation, UPF, Barcelona 08003, Spain; 5 Current address: Program of Virology, Division of Medical Sciences, Harvard Medical School, Boston, MA 02115, USA

## Abstract

**Background:**

Most odors are perceived to have the same quality over a large concentration range, but the neural mechanisms that permit concentration-invariant olfactory perception are unknown. In larvae of the vinegar fly *Drosophila melanogaster*, odors are sensed by an array of 25 odorant receptors expressed in 21 olfactory sensory neurons (OSNs). We investigated how subsets of larval OSNs with overlapping but distinct response properties cooperate to mediate perception of a given odorant across a range of concentrations.

**Results:**

Using calcium imaging, we found that ethyl butyrate, an ester perceived by humans as fruity, activated three OSNs with response thresholds that varied across three orders of magnitude. Whereas wild-type larvae were strongly attracted by this odor across a 500-fold range of concentration, individuals with only a single functional OSN showed attraction across a narrower concentration range corresponding to the sensitivity of each ethyl butyrate-tuned OSN. To clarify how the information carried by different OSNs is integrated by the olfactory system, we characterized the response properties of local inhibitory interneurons and projection neurons in the antennal lobe. Local interneurons only responded to high ethyl butyrate concentrations upon summed activation of at least two OSNs. Projection neurons showed a reduced response to odors when summed input from two OSNs impinged on the circuit compared to when there was only a single functional OSN.

**Conclusions:**

Our results show that increasing odor concentrations induce progressive activation of concentration-tuned olfactory sensory neurons and concomitant recruitment of inhibitory local interneurons. We propose that the interplay of combinatorial OSN input and local interneuron activation allows animals to remain sensitive to odors across a large range of stimulus intensities.

## Background

Sensory information varies in two major dimensions – quality and quantity. For our perception of the external world to be stable and useful, the brain must construct a relatively consistent percept of quality independent of quantity. At extremes of input quantity, concentration-invariance of stimulus quality fails. In vision, colors lose their salience at low luminance, while very high luminance can blind the visual system. In olfaction, faint odors just above the sensory threshold often lack any semantically accessible quality, while high odor concentrations can take on an irritating quality [1]. Aside from these extremes of input quantity, sensory systems retain a remarkably stable percept of quality across a large range of sensory input quantity [2].

Concentration-invariant quality perception is a general feature of olfactory systems [3-5]. Imaging studies in insects and vertebrates have noted that new olfactory glomeruli are sequentially recruited with increasing odor concentrations, probably reflecting the progressive activation of lower-affinity odorant receptors (ORs) with increasing odor concentrations [6-11]. How perceived odor quality is held stable even in the face of concentration-dependent changes in the spatial odor code is unknown [12,13]. Physiological analysis of early olfactory processing has documented that both presynaptic and postsynaptic inhibition mechanisms can shape olfactory information [14-21]. Whether these inhibitory interactions are used to modulate odor perception and behavior has received little experimental attention [21].

We investigated the problem of concentration-invariant olfactory behavior in the larval stage of the vinegar fly *Drosophila melanogaster*, which is an ideal system to examine this question because it has a miniaturized olfactory system with 21 pairs of olfactory sensory neurons (OSNs) expressing a combination of 25 ORs [22-24] and shows robust and easily measured odor-evoked behaviors [22,25,26]. Using calcium imaging, we developed a novel preparation to characterize the native response profile of larval OSNs. The response profiles we obtained agree qualitatively with previously reported ligand tuning of larval ORs ectopically expressed in the adult 'empty neuron' system [23,27,28]. Importantly, we found that only three larval OSNs showed reliable responses to ethyl butyrate. Using quantitative analysis of larval chemotactic behavior in defined odor environments [26,29], we studied the contribution of individual OSNs to the perception of ethyl butyrate. Although individual OSNs sufficed for behavior at distinct odor concentrations, the wild-type combination of 21 functional OSNs was necessary for individuals to display attraction across a 500-fold range of concentrations. Analysis at three levels of the larval olfactory system showed that inhibitory local interneurons (LNs) are not activated at low odor concentrations, but are recruited by the summed activation of multiple OSNs. The progressive activation of OSNs optimized for different concentration ranges, combined with the selective activation of inhibitory LNs at high odor concentrations, constitutes an elegant solution for maintaining consistent attraction to odors across a wide range of stimulus intensity.

## Results

### Odor ligand tuning of individual larval olfactory sensory neurons

**Figure 1:**
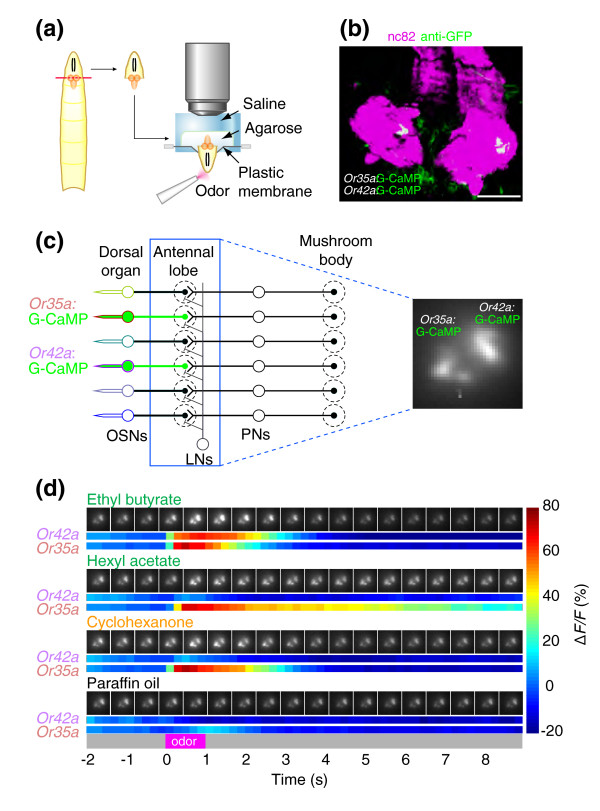
Imaging odor-evoked activity in larval olfactory neurons. **(a) **Schematic of the larval imaging preparation showing head dissection (left) and mounting of inverted sample for G-CaMP imaging (right). **(b) **Whole-mount immunofluorescence staining of G-CaMP in terminals of *Or35a *and *Or42a *OSNs (anti-GFP; green) counterstained with the neuropil marker nc82 (magenta). Confocal image is a flattened z-stack of 7 × 7.2 μm optical slices that covers the anterior portion of the larval brain neuropil oriented with anterior at bottom. Scale bar = 50 μm. Genotypes for this and all other strains used in the paper are listed in the Additional data file 1. **(c) **Schematic of the larval olfactory circuit of the animal in (b). Olfactory sensory neuron (OSN) activity is imaged at axon terminals in the antennal lobe (blue box). Glomeruli also receive input from local interneurons (LNs) and projection neurons (PNs). Intrinsic G-CaMP fluorescence of OSN axon termini viewed in the imaging setup (right). **(d) **Calcium dynamics of *Or35a *and *Or42a *OSNs in a single animal in response to three odorants (10^-2 ^odor dilution) and paraffin oil (solvent). For each stimulus, raw gray-scale fluorescent images presented at 600-ms intervals are shown at the top and false color-coded time traces represented by Δ*F*/*F *(%) (scale at right) are shown at the bottom. Odor presentation (1 s) is indicated in magenta on the gray time axis at the bottom.

To examine the ligand tuning of individual larval OSNs, we developed a preparation to image odor-evoked calcium increases at axon terminals of genetically labeled neurons (Figure 1a). The Gal4-UAS system [30] was used to express the genetically encoded calcium sensor, G-CaMP [31], in identified larval OSNs using Gal4 drivers with promoters from individual larval OR genes [22] (Figure 1b,c). We observed robust odor-evoked fluorescence increases in the axon terminals of larval OSNs. An example of odor-evoked calcium signals from two OSNs expressing *Or35a *and *Or42a *is shown in Figure 1d. Three different odors differentially activated these two OSNs. Ethyl butyrate activated both neurons, but hexyl acetate and cyclohexanone selectively activated only *Or35a *(Figure 1d). The response duration in a given OSN was odor-dependent. For instance, hexyl acetate induced a prolonged response in the *Or35a*-expressing OSN but cyclohexanone elicited a shorter response in the same neuron (Figure 1d).

**Figure 2:**
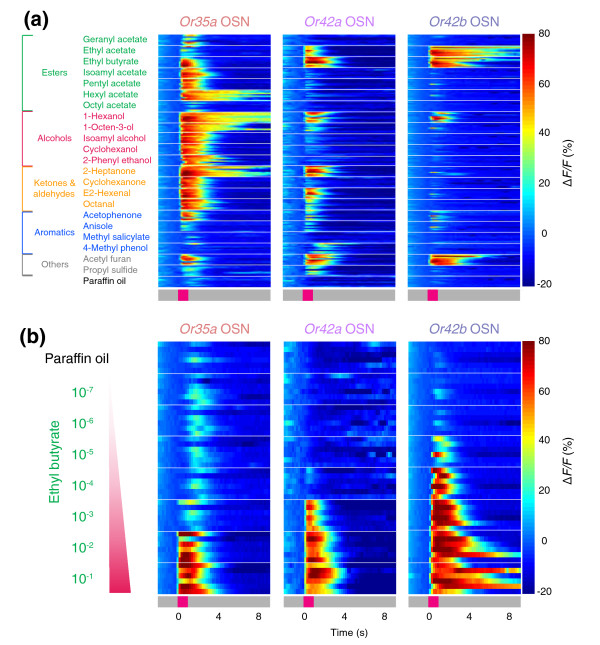
Ligand tuning of larval olfactory neurons in wild-type animals. **(a) **Odor-response profiles of the three OSNs most sensitive to ethyl butyrate, measured at axon termini of a given OSN in the antennal lobe, against a panel of 22 odorants (10^-2 ^odor dilution) and paraffin oil (solvent). Responses are shown as described in Figure 1d. Chemical structures and categorization by functional group of 22 odorants are at top left. Traces from *n* = 7–9 animals per stimulus are stacked. **(b) **Responses of *Or35a*, *Or42a*, and *Or42b *OSNs to an ethyl butyrate concentration series and paraffin oil (solvent) represented as Δ*F/F *(%) (scale at right). Traces from *n* = 6–8 animals per genotype and stimulus are stacked.

We applied this imaging technique to examine the native responses of 11 larval OSNs to a panel of 22 odors (Figure 2a and Additional data file 1 (Figure S1a)). The ligand selectivity of larval OSNs we tested varied widely. *Or35a*-expressing OSNs reliably responded to 15/22 odors, while *Or82a*-expressing neurons responded only to geranyl acetate. Our results match the response profile of larval ORs studied by ectopic expression in the adult 'empty neuron' system [23,27,28]. Consistent with previous reports, larval OSNs could be categorized into aromatic odor-sensitive and non-aromatic odor-sensitive classes (Additional data file 1 (Figure S1b,c)) [23].

Among the panel of odors tested, we focused on ethyl butyrate, an ester widely found in fruits [32] and thus likely to be encountered by larvae in their natural habitat. *Drosophila *larvae show robust chemotaxis to this ester [22]. Our calcium-imaging results indicated that ethyl butyrate consistently activated only 3 of the 11 larval OSNs we tested: *Or35a*, *Or42a*, and *Or42b *(Figure 2a and Additional data file 1 (Figure S1a)). None of the other ten remaining larval ORs responded strongly to ethyl butyrate in previous studies [23,28]. Thus, we conclude that these three OSNs constitute the primary sensors of ethyl butyrate in the larval olfactory system. Because G-CaMP imaging lacks the sensitivity and temporal resolution of electrophysiology, we cannot exclude the possibility that other neurons are weakly activated by ethyl butyrate, but below the detection threshold of G-CaMP. Hoare *et al*. [33] recently reported stochastic ('fuzzy') electrophysiological responses to odor stimulation in various larval OSNs, but did not examine responses of *Or35a*-, *Or42a*-, or *Or42b*-expressing neurons to ethyl butyrate. All three OSNs responded reliably to odors in our imaging study. Therefore, we did not find evidence supporting the fuzzy nature of the odor code reported for other larval OSNs.

### Concentration-dependent responses in OSNs to ethyl butyrate

To ask whether *Or35a*, *Or42a*, and *Or42b *OSNs show differential sensitivity to ethyl butyrate, we carried out a dose-response analysis of these OSNs by calcium imaging. Whereas all three OSNs responded to high concentrations of ethyl butyrate (10^-2 ^dilution of odor (v:v in paraffin oil), referred to henceforth as 'odor dilution'; Figures 1d and 2a), the odor concentration threshold at which these OSNs first reliably responded differed greatly (Figure 2b). *Or35a *OSNs showed reliable responses only at the 10^-2 ^odor dilution, *Or42a *OSNs had a response threshold of 10^-3 ^odor dilution, and *Or42b *OSNs responded initially at 10^-4 ^odor dilution.

We assessed the stability of these differential odor thresholds in wild-type larvae having 21 functional neurons compared with those obtained from larvae that had only a single functional OSN. Larvae with a single functional OSN were constructed by exploiting the *Or83b *mutation, which renders animals insensitive to odors by preventing the normal trafficking and functioning of all OR proteins [34,35]. By genetically restoring wild-type *Or83b *function to individual neurons using the Gal4-UAS system [30], we restored normal OR trafficking and function only in a given OSN [22,26]. Such genetically manipulated animals, which we term '*OrX*-functional', were constructed in this study by restoring *Or83b *function either to *Or35a*, *Or42a*, or *Or42b *OSNs in anosmic *Or83b*^-/- ^mutants. There was no statistically significant difference between the sensitivity of wild-type and *OrX*-functional OSNs to ethyl butyrate (Figure 2b and Additional data file 1 (Figures S2 and S3); see also EC_50 _values in Materials and methods). This suggests that pre-synaptic inhibition reported for the adult olfactory system in flies and vertebrates is unlikely to play a critical role in larvae [15,20,21,36].

### Behavioral sensitivity to ethyl butyrate in wild-type and manipulated larvae

**Figure 3:**
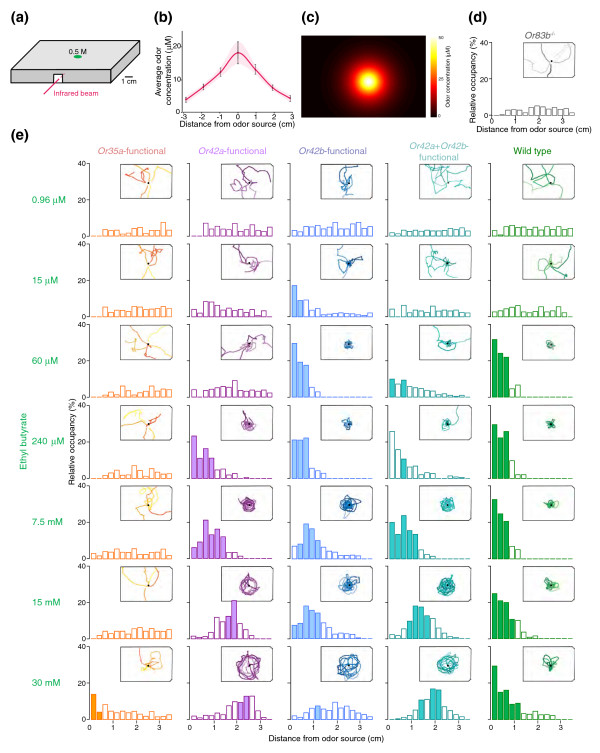
Behavioral sensitivity to ethyl butyrate in wild-type and manipulated larvae. **(a) **Schematic of the single odor source assay, with a 0.5 M ethyl butyrate source at position E7 on the lid of a 96-well plate used to generate a radial odor gradient. **(b) **Average odor concentrations in gaseous phase (μM) obtained by Fourier transform-infrared (FT-IR) spectroscopy along the length of the arena shown in (a). Odor concentrations (mean ± SEM) were measured 1–5 minutes after loading. **(c) **Topographic reconstruction of the single odor source gradient shown in (b). **(d) **Behavior of *Or83b*^-/- ^larvae in the single odor source assay. Inset shows merged locomotor tracks for *n* = 5 animals, acquired consecutively, with the position of the ethyl butyrate source (60 mM) indicated by the black dot. Bar plots show the median relative occupancy with respect to the distance to odor source (*n* = 15 larvae). See Materials and methods for details on how occupancy distributions were calculated and evaluated with non-parametric tests for statistical significance. For clarity in data presentation, we have omitted the interquartile distances from this figure. **(e) **Odor-evoked behavior of wild-type and *Or35a*-, *Or42a*-, *Or42b*-, *Or42a*+*Or42b*-functional larvae in the single odor source assay for increasing source concentrations of ethyl butyrate (n = 15 larvae per genotype and stimulus) plotted as described in (d). Bins of relative occupancy that differ significantly from *Or83b*^-/- ^are shaded (Wilcoxon test; corrected *p *< 0.0036). The first two bars of *Or42a*+*Or42b*-functional larvae tested at 240 μM are unshaded because large fluctuations around the mean make these data not significantly different from *Or83b *mutants.

**Figure 4:**
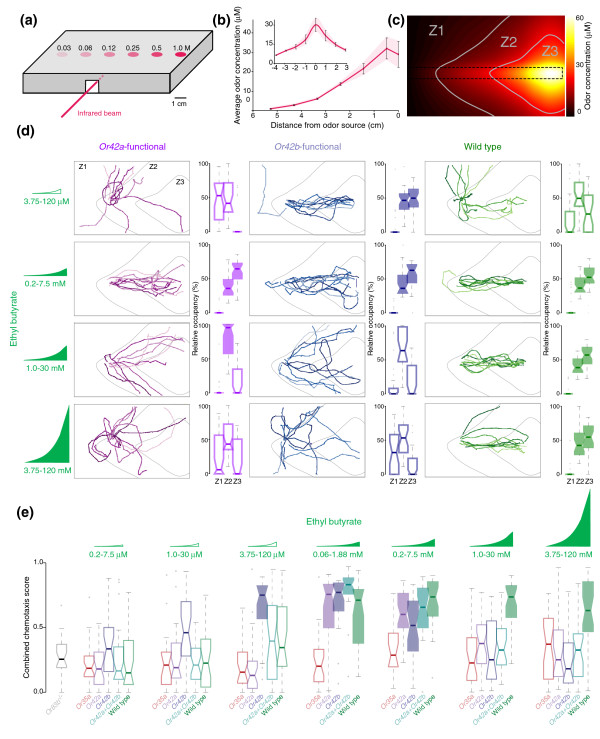
Chemotaxis to ethyl butyrate in wild-type and manipulated larvae. **(a) **Schematic of the multiple odor source assay. Source concentrations (M) used to generate ethyl butyrate gradients. **(b) **Average odor concentrations (mean ± SEM) obtained by FT-IR for sections along the length and the width (inset) of the arena shown in (a). Odor concentrations (mean ± SEM) were measured 4–12 minutes after loading. **(c) **Topographic reconstruction of the multiple odor source gradient shown in (b). The odorant line is indicated by the dashed box. The arena was subdivided into three concentration zones indicated by the gray lines (Z1 = low (0–8.7 μM), Z2 = medium (8.7–24.2 μM), and Z3 = high (24.2–60 μM)). **(d) **Odor-evoked behavior of *Or42a*-functional (left), *Or42b*-functional (middle) and wild-type (right) larvae tested in the multiple source assay. Source concentration range is indicated at the left. Gradient cartoons are not to scale and represent the relative concentration differences between the gradients. Low-concentration gradients are indicated with open gradient symbols and high concentration gradients with filled gradient symbols. Ten merged tracks, acquired consecutively, are shown per genotype and stimulus. Percentages of time in zones Z1–Z3 are represented at the right of the tracks as boxplots (n = 30), in which the boundaries represent first and third quartiles, the 'waist' indicates the median, whiskers are 1.5 interquartile distance, and outliers are marked with gray dots. Data that differ significantly from *Or83b*^-/- ^(source range: 3.75–120 mM) are shaded (Wilcoxon test; corrected *p *< 0.0056). **(e) **Quantification of the overall alignment of trajectories with the gradient (n = 30 for all genotypes, except for *Or35a*-functional *n* = 20–30). Data that differ significantly from *Or83b*^-/- ^(source range: 3.75–120 mM; gray boxplot at left) are shaded (Wilcoxon test; corrected *p *< 1.4 × 10^-4^).

The differential sensitivity to ethyl butyrate of *Or35a*, *Or42a *and *Or42b *OSNs prompted us to ask whether these three OSNs mediate concentration-dependent behavioral responses to ethyl butyrate. To investigate this question we used two different experimental paradigms, which measure different aspects of olfactory behavior. A single odor source device [26] (Figure 3) was used to quantify the olfactory sensitivity of individual larvae to a point source of an odor, and a multiple odor source device [26] (Figure 4) was used to assess the ability of larvae to ascend odor gradients.

In the single odor source assay, a drop of ethyl butyrate of desired concentration was introduced into the lid in the center of a rectangular arena (Figure 3a). Diffusion of odorant molecules generated a Gaussian-like radially symmetric odor distribution centered on the source [26] (Figure 3b). Odor concentrations in air were considerably lower than source concentrations (compare 500 mM source with 50 μM peak gradient; Figure 3a–c). Single larvae were introduced into the arena under a drop of ethyl butyrate of varying concentrations ('the odor source'), and their position was tracked for 5 minutes. We observed three different responses to odors in this assay, which allowed us to classify the olfactory sensitivity of our larvae. Animals that can detect the odor, and are attracted to it, will remain in close proximity to the odor source. Animals that do not detect the odor, such as the anosmic *Or83b *mutants, dispersed in the arena (Figure 3d). Finally, animals that can detect the odor but are repelled by the high concentration rapidly leave the area under the point source and navigate in isoconcentration circles at a distance from the source.

To quantify odor responses in this assay, the spatial distribution of each animal within a set of concentric 0.25 cm circles was determined. Because anosmic *Or83b*^-/- ^control larvae dispersed in the arena (tracks in inset in Figure 3d) and showed a flat occupancy distribution (bar plot histogram, Figure 3d), we defined dispersion as a failure to detect the odor, and remaining in proximity to the odor as odor detection.

At low source concentrations of ethyl butyrate (0.96 μM or 15 μM), the distribution of wild-type larvae did not differ significantly from that of *Or83b*^-/- ^control larvae (Figure 3e, green). However, at concentrations of 60 μM and 240 μM, wild-type larvae remained within less than 1 cm of the odor source throughout the 5-minute experiment (Figure 3e, green). The attraction of wild-type larvae to ethyl butyrate was remarkably stable, such that animals remained within approximately 1 cm of even very high source concentrations ranging from 7.5 to 30 mM (Figure 3e, green). We conclude that the olfactory threshold of wild-type larvae to ethyl butyrate is 60 μM and that these animals have a mechanism to remain attracted to this odor over at least a 500-fold concentration range. We propose that this consistent attraction to a point source of odor that varies across a wide range of concentrations is evidence for concentration-invariant behavior by wild-type larvae.

To ask whether concentration-invariant attraction requires combinatorials of functional OSNs, we examined the sensitivities of larvae with olfactory input limited to a single OSN expressing *Or35a*, *Or42a*, or *Or42b*. Consistent with the low ethyl butyrate sensitivity of the *Or35a *OSN, *Or35a*-functional animals did not show any behavioral responses to ethyl butyrate between 0.96 μM and 15 mM, but showed weak, yet significant, behavioral responses to a high concentration of ethyl butyrate (30 mM; Figure 3e, orange).

*Or42a*-functional animals were less sensitive to ethyl butyrate than wild-type larvae, showing a threshold sensitivity of 240 μM (Figure 3e, violet). As odor concentrations increased, *Or42a*-functional larvae showed a characteristic circling behavior in which they occupied a circle of increasing diameter from the odor source, ranging as odor concentrations increased from 1 cm with a 240 μM odor source to 2.25 cm with a 30 mM odor source (Figure 3e, violet).

Larvae with the high-sensitivity *Or42b *OSN were more sensitive to odors than wild-type larvae, showing a significant response to 15 μM ethyl butyrate (Figure 3e, blue), a source concentration at which wild-type larvae show no odor responses (Figure 3e, green). Like *Or42a*-functional larvae, *Or42b*-functional larvae showed concentration-dependent circling behavior and increased their distance from the source as ethyl butyrate concentrations increased.

The effect of summed OSN input on concentration-dependent olfactory behavior was measured in *Or42a*+*Or42b *'double' OSN functional larvae. Their odor sensitivity threshold was 60 μM, intermediate between that of *Or42a*-functional larvae and *Or42b*-functional larvae. *Or42a*+*Or42b*-functional larvae also showed the circling behavior characteristic of the single functional strains (Figure 3e, cyan).

From an examination of the temporal evolution of the mean distance to odor over the 5-minute experiment (Additional data file 1 (Figure S4)), we can confirm that larvae with one or two functional OSNs are circling at a distance because they are actively repelled by high odor concentrations under the odor source. At the same time, we can exclude the alternative explanation that these manipulated larvae fail to detect an increase in the odor concentration because of sensory neuron saturation. With a 15 mM ethyl butyrate source, *Or83b *mutants left the source of the odor immediately and spent the rest of the 5-minute period exploring the plate. In contrast, wild-type larvae initially moved away from this odor stimulus but within 60 s of exploration at up to 1 cm away from the point source, these animals returned and stayed within about 0.5 cm of the odor source for the balance of the 5-minute experiment. *Or42a*-functional animals showed the same departure and return behavior. However, they overshot their preferred distance (approximately 2 cm from the odor source) and returned to it afterwards without visiting the region under the source. They never returned to their original location under the odor source. This strongly argues that single OSN-functional larvae are repelled by high concentrations of odor located close to the point source.

Genetic manipulation of the larval olfactory system to reduce input to one or two OSNs thus dramatically changes the animal's behavior to ethyl butyrate across a large concentration range. Single-OSN- and double-OSN-functional larvae lost the ability to maintain consistent attraction to ethyl butyrate across the concentrations tested and instead showed increasing avoidance of the odorant as concentrations increased. For technical reasons, we were unable to compare the absolute odor concentrations used in calcium imaging with those used in behavior, but in both experimental paradigms *Or42b *was about 10 times more sensitive than *Or42a *and 100 times more sensitive than *Or35a*.

### Chemotaxis to ethyl butyrate in wild-type and manipulated larvae

To test further the ability of individual ethyl butyrate-sensitive OSNs to detect subtle changes in odor concentrations, we challenged single-OSN-functional animals in a multiple odor source assay [26] (Figure 4a–c). This assay differs from that in Figure 3 because animals start at the low concentration end of the gradient rather than being placed directly under the highest odor concentration as in the single odor source assay. The assay tests the ability of larvae to detect and ascend odor gradients. An exponential gradient of ethyl butyrate was created based on six odor sources aligned in the middle of the arena (Figure 4a) and validated by infrared spectroscopy (Figure 4b). We arbitrarily divided the arena into three zones of low (Z1), medium (Z2), and high (Z3) ethyl butyrate concentrations (Figure 4c) defined on the basis of concentration isoclines of the gradient. Single larvae were introduced into the assay at the low end of the gradient and their movement tracked as described elsewhere [26,29]. The percentage time that each animal spent in zones Z1–Z3 was calculated (Figure 4d). The ability of individual larvae to follow the odorant line was quantified with a combined chemotaxis index [26] (Figure 4e). Chemotaxis was studied in ethyl butyrate gradients of varying amplitude.

*Or83b*^-/- ^mutant larvae did not chemotax in the highest concentration range of ethyl butyrate gradient (3.75–120 mM; Figure 4e, gray boxplot). *Or35a*-functional larvae did not chemotax in response to any gradients tested (Figure 4e, orange boxplots). The failure of *Or35a*-functional larvae to chemotax may be because the starting concentration of all gradients tested here was below the high detection threshold of these low-sensitivity animals.

In gradients ranging from low (0.2–7.5 mM) to high concentrations (3.75–120 mM), wild-type larvae showed consistently strong chemotaxis, characterized by spending significantly more time in medium to high concentration zones (Z2–Z3; Figure 4d, green) and by a high combined chemo-taxis score (Figure 4e, right). Thus, the same concentration-invariant olfactory behavior of wild-type larvae seen in the single odor source assay (Figure 3) was obtained in the multiple odor source chemotaxis assay.

In contrast, *Or42a*-functional animals showed robust chemotaxis over a narrower concentration range of 0.06–1.88 mM to 0.2–7.5 mM and only showed significant accumulation in the high-concentration Z3 zone in the 0.2–7.5 mM gradient (Figure 4d, magenta). As gradient concentrations increased, these animals showed a characteristic avoidance of the high-concentration Z3 zone and instead accumulated in the intermediate Z2 zone (Figure 4d, magenta). When odor concentrations increased further, these animals lost all ability to chemotax and did not differ from *Or83b*^-/- ^mutants in their combined chemotaxis score (Figure 4e).

*Or42b*-functional larvae showed strong chemotaxis behavior at considerably lower concentrations than wild-type larvae (3.75–120 μM gradient; Figure 4d, blue). Like *Or42a*-functional larvae, they avoided the high-concentration Z3 zone as gradient amplitudes increased and, unlike wild-type larvae, they failed to chemotax in gradients with the two highest amplitudes (Figure 4e).

### Odor-evoked responses at projection neuron terminals in the mushroom body

**Figure 5:**
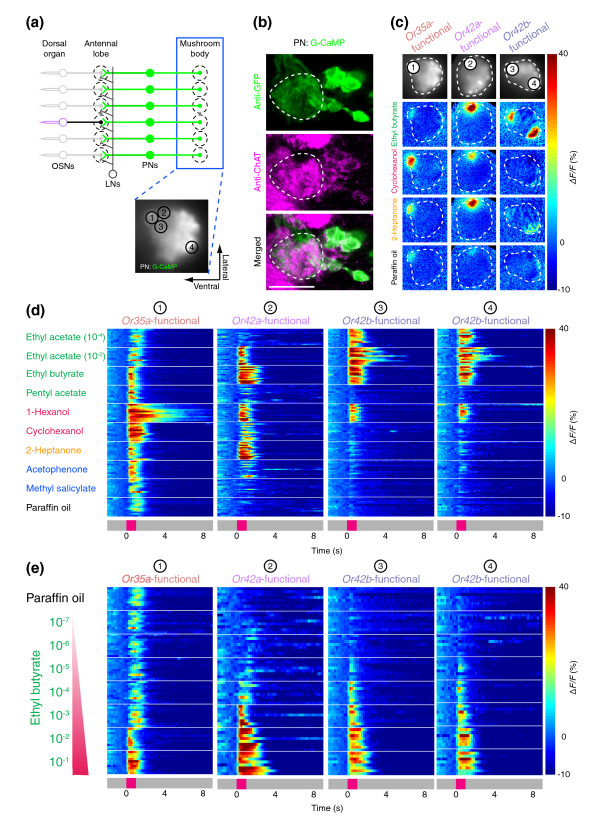
Odor responses of larval projection neurons. **(a) **Schematic for measuring functional activation of larval PNs in *Or35a*-, *Or42a*-, or *Or42b*-functional larvae at axon terminals in the mushroom body (blue box). Intrinsic G-CaMP fluorescence of the mushroom body, with subdomains 1–4 and sample orientation indicated (bottom). **(b) **Confocal image (flattened z-stack of 2 × 1.2 μm optical slices) of PN cell bodies stained to reveal G-CaMP (anti-GFP antibody, green) and *Drosophila *choline acetyltransferase (anti-ChAT, magenta). Scale bar = 20 μm. **(c) **Representative G-CaMP activity in PN terminals in mushroom body elicited by three odorants (10^-2 ^dilution) and paraffin oil (solvent) in (left to right): *Or35a*-, *Or42a*-, and *Or42b*-functional larvae. Top row shows intrinsic mushroom body G-CaMP fluorescence and bottom four rows show false color-coded image of mushroom body taken 600 ms after stimulus onset, and represented as %Δ*F*/*F *(scale at the right). **(d) **Responses of PNs of single-functional larvae in (b) to eight odorants (10^-2 ^dilution except as indicated) and paraffin oil (solvent) represented as false color-coded time traces (%Δ*F*/*F*; scale at bottom right). Traces from *n* = 11–14 animals per stimulus are stacked. Region of analysis is from major subdomain 1–4, as indicated in (a-b). **(e) **Responses of major subdomains 1–4 of PN axon termini in mushroom body of *Or35a*-, *Or42a*-, and *Or42b*-functional larvae to an ethyl butyrate concentration series and paraffin oil (solvent) represented as Δ*F*/*F *(%) (scale at right). Traces from *n* = 8 animals per genotype and stimulus are stacked.

To examine how input from three ethyl butyrate-sensitive OSNs – *Or35a*, *Or42a*, and *Or42b *– is relayed to higher olfactory centers, we imaged odor-evoked responses at projection neuron (PN) axon terminals in the mushroom body (Figure 5a). GH146, a Gal4 driver that labels the majority of larval PNs [37], was used to drive G-CaMP for calcium imaging in the axon terminals of PNs in the mushroom body (Figure 5a). Larval GH146-expressing PNs are cholinergic (Figure 5b), confirming previous analysis of adult PNs [38,39]. In initial experiments, we attempted to image PN responses in wild-type larvae having 21 functional OSNs. Unfortunately, insufficient spatial resolution and the absence of PN-specific genetic markers produced inconclusive results (data not shown). Imaging signals can be obtained in response to odor stimulation, but we have no means of mapping the resulting data onto a coordinate system for a given PN. To solve this registration problem, olfactory input was genetically restricted to a single olfactory neuron by carrying out imaging in *Or35a*-, *Or42a*-, or *Or42b*-functional larvae. A representative subset of eight odors was used to probe responses in PNs.

Odors activated distinct single and positionally conserved mushroom body glomeruli in both *Or35a*- and *Or42a*-functional animals (Figure 5c). In *Or42b*-functional animals, odors reliably activated two mushroom body glomeruli (Figure 5c). This observation could be due to terminal axonal branching of a single PN innervating the *Or42a *OSN or to two PNs innervating the *Or42b *OSN, but was not investigated further here. In some cases there was faint activation outside of the primary glomeruli analyzed here (Additional data file 1 (Figure S5)), but we focused our analysis on the most reliably and strongly activated regions in the mushroom body. These data comprise the first report of odor-evoked responses in the larval mushroom body. Importantly, our results provide functional confirmation of previous anatomical analysis showing that the larval mushroom body is organized into discrete glomeruli representing a 1:1 synaptic relationship between OSNs and PNs in the olfactory circuit [37].

Analysis of PN responses to a panel of eight odors in the engineered configuration of input from only a single OSN revealed a good qualitative correspondence between the response profile of the primary olfactory neurons and second-order PNs (compare Figures 5d and 2b). The only exception was cyclohexanol, which did not significantly activate the *Or42a *OSN, but did elicit a weak response in PN terminals in the mushroom body of *Or42a *functional animals. Consistent with previous observations of *Or42a *and *Or42b *receptor tuning made in the empty neuron system [28], the PN response to ethyl acetate was strongly concentration dependent for *Or42a*- and *Or42b*-functional animals. Whereas both *Or42a *and *Or42b *PNs showed responses at a 10^-2 ^dilution of ethyl acetate, only *Or42b *responded to a 10^-4 ^dilution of ethyl acetate (Figure 5d). Direct quantitative comparisons of the thresholds of PNs and OSNs are not possible because different versions of G-CaMP were used to image these cells, but we note that for both cell types, *Or42b *was about 10 times more sensitive than *Or42a *and 100 times more sensitive than *Or35a *(Figure 5e; see EC_50 _values of PNs in Materials and methods).

### High-concentration threshold for activation of inhibitory local interneurons

**Figure 6:**
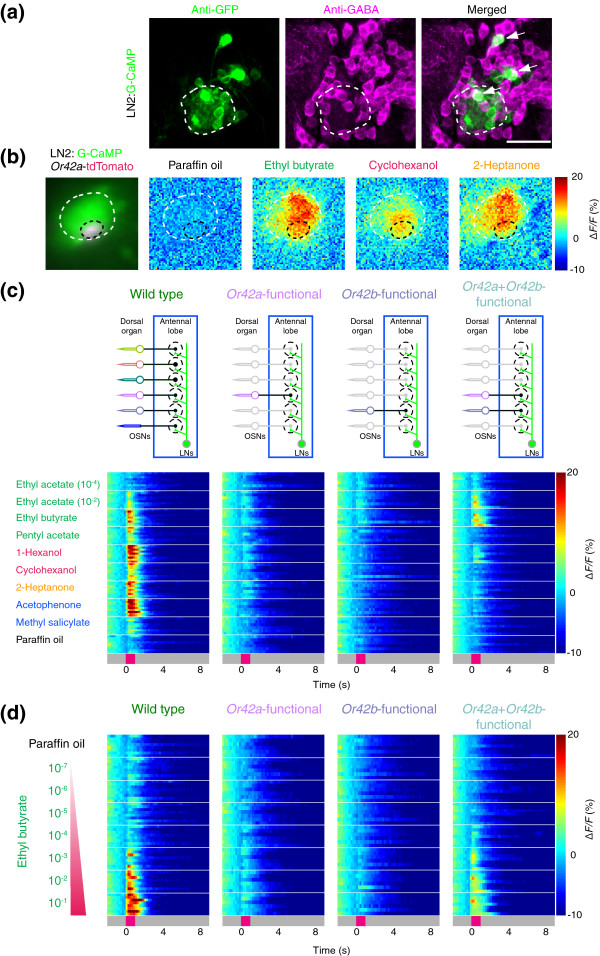
Threshold response properties of larval local interneurons. **(a) **LN2 cells in the antennal lobe stained to reveal G-CaMP (left; anti-GFP antibody, green) and gamma-aminobutyric acid (middle; anti-GABA, magenta). Arrows in the merged image (right) indicate GABA-positive LN2 neurons. **(b) **Imaging LN activation at the terminal of the *Or42a *neuron, as marked by an *Or42a*-nsyb:tdTomato reporter. The leftmost panel is a merged image of intrinsic fluorescence of G-CaMP (green) and nsyb::tdTomato (magenta). The boundary of the antennal lobe is marked with a white dashed line and the *Or42a *glomerulus with a black dashed line. The other three panels show antennal lobe calcium responses to paraffin oil (solvent) and three odorants (10^-2 ^dilution) taken 400 ms after stimulus onset, represented as Δ*F*/*F *(%) (scale at right). **(c) **Top panel: schematic for measuring functional activation of LN2 neurons in the antennal lobe (blue boxes) of wild-type, *Or42a*-, *Or42b*-, and *Or42a*+*Or42b*-functional larvae. Bottom panel: responses of LN2 neurons in larvae of indicated genotype to eight odorants (10^-2 ^dilution except as indicated) and paraffin oil (solvent) represented as false color-coded time traces (%Δ*F*/*F*; scale at bottom right). Traces from *n* = 6–9 animals per stimulus are stacked. **(d) **Responses in LNs in larvae of indicated genotype to an ethyl butyrate concentration series and paraffin oil (solvent) represented as Δ*F*/*F *(%) (scale at right). Traces from *n* = 6–8 animals per genotype and stimulus are stacked.

Inhibitory LNs in the adult insect antennal lobe have been implicated as modulators of olfactory information processing [17,18,38], but no functional analysis of larval *Drosophila *LNs has been described. To image odor-evoked activation of larval LNs, we characterized the expression patterns of Gal4 lines known to be expressed in LNs in the adult antennal lobe (Additional data file 1 (Figure S6)). Of the four Gal4 lines tested, only LN2-Gal4 [40] selectively labeled LNs that were positive for the inhibitory neurotransmitter gamma-aminobutyric acid (GABA) and negative for choline acetyltransferase, a marker of cholinergic neurons (Figure 6a and Additional data file 1 (Figure S6a)). The LNs labeled by LN2-Gal4 extended processes throughout all glomeruli in the larval antennal lobe (Figure 6a), consistent with previous descriptions of larval LN connectivity [37].

Unlike the glomerulus-specific activation patterns evoked by activation of OSNs, odors induced global activation of LN processes throughout the antennal lobe (Figure 6b). To standardize our analysis of LN responses, we restricted the area of interest to genetically labeled terminals of the *Or42a *OSN in the antennal lobe (Figure 6b, left panel) and used eight representative odors to probe LN activation in wild-type larvae and single- and double-OSN-functional larvae (Figure 6c). LNs in wild-type larvae responded strongly and reliably to only four of the eight odors: ethyl butyrate, 1-Hexanol, 2-Heptanone, and acetophenone. Weak responses were found for a 10^-2 ^dilution of ethyl acetate, pentyl acetate, cyclohexanol, and methyl salicylate. No responses were detected after application of a 10^-4 ^dilution of ethyl acetate. When we restricted olfactory input to the *Or42a *or *Or42b *neurons only, the LNs did not respond to any of the odors tested. Larvae in which both the *Or42a *and *Or42b *neurons were functional showed weak responses to a 10^-2 ^dilution of ethyl acetate, ethyl butyrate, and 1-Hexanol and no responses to the remaining five odors (Figure 6c).

These results suggest that the LNs may have a higher odor-activation threshold than OSNs or PNs, and further that summation of OSN input modulates LN responses. To explore this idea, we asked how LNs respond to ethyl butyrate in a range of odor dilutions from 10^-1 ^to 10^-7^(Figure 6d). In wild-type larvae, LNs showed reliable responses only at 10^-2 ^and 10^-1 ^dilutions of ethyl butyrate, with partial activation at 10^-3 ^odor dilution. LNs of *Or42a *or *Or42b *single functional animals did not respond to any concentration of ethyl butyrate, but the summed input of *Or42a *and *Or42b *neurons in *Or42a*+*Or42b*-functional neurons induced modest responses of LNs from 10^-1 ^to 10^-3 ^dilutions of ethyl butyrate only (Figure 6d).

### Inhibition of PN odor responsivity by summed OSN input

**Figure 7:**
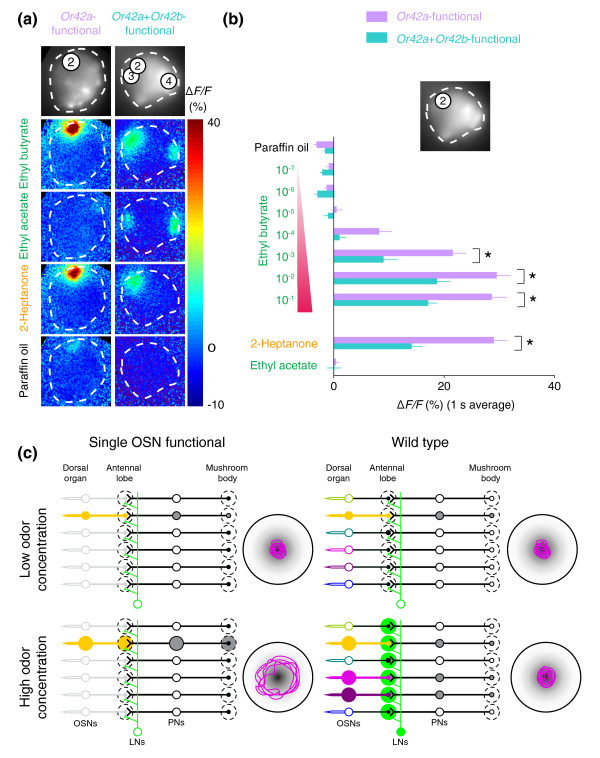
Modulation of odor-evoked signals in the mushroom body by addition of a second functional OSN. **(a) **Representative G-CaMP activity in PN terminals in mushroom body elicited by three odorants (10^-2 ^dilution except ethyl acetate, 10^-4 ^dilution) and paraffin oil (solvent) in *Or42a*+*Or42b*-functional larvae compared to *Or42a*-functional larvae (all but the ethyl acetate image are reprinted from Figure 5c). Top image shows intrinsic mushroom body G-CaMP fluorescence with overlaid numbers indicating the location of subdomains in Figure 5a. Bottom four images show false color-coded image of mushroom body taken 600 ms after stimulus onset, and represented as Δ*F*/*F *(%) (scale at the right). **(b) **Responses of subdomain 2 to high concentrations of ethyl butyrate are decreased in *Or42a*+*Or42b*-functional larvae compared to those in *Or42a*-functional larvae. Responses to a dilution series of ethyl butyrate, 2-Heptanone (10^-2 ^odor dilution), ethyl acetate (10^-4 ^dilution), and paraffin oil are calculated as the average Δ*F*/*F *over 1 s after odor stimulus onset (mean ± SEM). Purple: *Or42a*-functional larvae (n = 8). Light blue: responses from *Or42a*+*Or42b*-functional larvae, *n* = 5 except paraffin oil (n = 6), 10^-4 ^and 10^-2 ^dilutions of ethyl butyrate (n = 6), 10^-2 ^dilution of 2-Heptanone (n = 6), and 10^-4 ^dilution of ethyl acetate (n = 6). Responses that differ significantly between the two genotypes are indicated with an asterisk (*p < 0.01, Student's *t*-test). **(c) **Schematic model of gain control in the larval olfactory system. In single-OSN-functional animals (left), low concentrations of odor cause moderate activation of the single OSN and its PN, leading to attraction to the odor source (magenta trajectory to the right). High concentrations of odor fail to activate the LNs (green) and cause strong activation of the PN and corresponding behavioral avoidance of the odor. In wild-type animals, low odor concentrations activate a single OSN and its PN, leading to odor attraction. At high odor concentration, two additional OSNs are recruited and the LN network is activated, preventing PN activity from reaching saturation and maintaining stable attraction to the odor.

The recruitment of LN activation by summation of OSN input prompted us to ask if PN output is modulated according to the magnitude of OSN input. *Or42a*+*Or42b*-functional larvae were constructed to express G-CaMP under the control of GH146 and odor-evoked calcium activation was measured at PN terminals in the mushroom body as described in Figure 5. This was technically demanding because our CCD-based imaging system lacks the three-dimensional resolution to image odor-evoked calcium responses simultaneously at multiple Z planes. Thus, only samples in which the three activated mushroom body glomeruli in *Or42a*+*Or42b*-functional animals were fortuitously located in the same focal plane could be analyzed (Figure 7a). Between five and six samples with such an orientation were analyzed for responses to ethyl acetate, 2-Heptanone, and a concentration series of ethyl butyrate (Figure 7b). Responses in the *Or42a*-specific subdomain were compared with data obtained from the same subdomain in *Or42a*-functional animals in Figure 5c–e. For optimal comparisons across these genotypes, strains were designed such that the same insertion of *Or42a*-*Or83b *was used and G-CaMP dosage was kept constant. Therefore, we are confident that any functional differences are a product of the biology of the circuit.

PN responses to 10^-3^, 10^-2^, and 10^-1 ^dilutions of ethyl butyrate were significantly weaker in *Or42a*+*Or42b*-functional animals compared to responses in *Or42a*-functional animals (Figure 7b). To ask if this reduction in response was specific to the *Or42a *activation subdomain, we tested 2-Heptanone, which selectively activates *Or42a *but not *Or42b *OSNs (Figure 2a) [28]. Unexpectedly, responses to 2-Heptanone were reduced in the two OSN-functional backgrounds, even though we did not detect an increase in LN function in *Or42a*+*Or42b*-functional larvae compared to *Or42a*-functional larvae (Figure 6c). It is plausible that spontaneous activity or weak evoked responses from the *Or42b*-functional neuron can modulate the LNs and thus the circuit dynamics, but that this was below the detection threshold of G-CaMP in Figure 6. Future work examining the synaptic physiology of these PNs in relation to OSN and LN input will be crucial for understanding the functional relationships within this circuit, as has recently been accomplished in the adult antennal lobe [20,41,42].

## Discussion

In the work reported here, we have established a methodology to monitor odor-evoked neural activity at three levels of the olfactory circuit in *Drosophila *larvae using calcium imaging. We identified three OSNs, those expressing *Or35a*, *Or42a*, and *Or42b*, as the primary sensors of ethyl butyrate. By constructing larvae receiving sole olfactory input from each of these neurons, we showed that the behavioral sensitivity threshold of such larvae is directly related to the response thresholds of the OSNs. Wild-type larvae use these three OSNs to respond consistently to ethyl butyrate over exponential gradients varying 60-fold in amplitude. Animals with only one of these OSNs functional showed attraction in a narrower concentration range and repulsion at higher concentrations. We further found that these OSNs communicated with dedicated postsynaptic PNs, and most notably, only activated inhibitory LNs at high ethyl butyrate concentrations and when activation of two OSNs was summed. Finally, we provide initial evidence that summed OSN input inhibits PN output. This work provides the first demonstration that LN activity increases with the number of input channels. We propose a model in which summed activation of OSNs, and the LNs postsynaptic to them, is essential for animals to achieve concentration-invariant olfactory attraction to ethyl butyrate.

### Intensity coding with combination of OSNs

The combinatorial odor-coding hypothesis, in which multiple OSNs cooperate to mediate the perception of odors, was proposed nearly a decade ago [43]. The behavioral relevance of this hypothesis has been challenging to test in most organisms because of the sheer number of ORs and OSNs activated by even a single odorant. Working in the *Drosophila *larva, we have previously shown that odor-evoked behavior [22] and chemotaxis up an odor gradient [26] is possible with only a single functional OSN. Thus, odor detection and computation of increasing concentrations of an odor can be accomplished without combinatorial coding. However, we found that behaviors obtained by adding two functional OSNs to an otherwise nonfunctional olfactory system produced responses that were not a simple sum of behaviors mediated by single neurons alone [22]. We hypothesized that a fine balance of inhibitory [38,44] and excitatory [39] interactions within the antennal lobe might contribute to the nonadditive effects that we observed in our previous work.

In the present study we show that one or two OSNs are insufficient to sense and maintain invariant attraction to a given odor – ethyl butyrate – across a very wide range of concentrations. Yet wild-type larvae with 21 functional OSNs remained strongly attracted by sources ranging across a 500-fold range of concentrations in the single odor source assay and across a 60-fold range of gradients in the multiple odor source assay. Unexpectedly, we found that *Or42b*-functional larvae were significantly more sensitive to this ester than wild-type larvae at very low ethyl butyrate concentrations. This posed a puzzle because wild-type larvae possess a functional *Or42b *neuron and yet do not display any behavioral response to the odor at low concentrations.

Furthermore, animals with only a single functional *Or35a*, *Or42a*, or *Or42b *neuron showed much narrower attraction to a specific concentration of ethyl butyrate, which was correlated with the sensitivity of the OSN as measured by calcium imaging. *Or42a*-, *Or42b*-, and *Or42a*+*Or42b*-functional larvae showed a characteristic behavior in which they circled odor sources of higher concentrations at progressively larger diameters. This suggested a distortion in the concentration perception of these animals, such that odors that were perceived by wild-type larvae to be attractive were perceived by single-OSN-functional larvae to be aversive.

### Local interneurons may act as a gain control mechanism

In searching for a mechanism to explain the aberrant behavior of single-OSN-functional larvae at both low and high concentrations of ethyl butyrate, we investigated the functional properties of inhibitory LN2 interneurons, which are selectively activated by the summed stimulation of *Or42a *and *Or42b *OSNs by ethyl butyrate. Such activity-dependent activation of local inhibitory interneurons has previously been suggested in the vertebrate olfactory bulb [19], where recruitment of effective lateral inhibition required the correlated firing of mitral cells tuned to the same odor. This study provides the first direct evidence that olfactory LNs are engaged depending on summed activities of OSNs.

Our observations are compatible with a model in which the LN2 neurons act as a gain-control mechanism for the olfactory circuit, as has recently been suggested on the basis of electrophysiological studies in the adult fly [20,21] (Figure 7c). We propose that in animals with a fully functional olfactory system, spontaneous activity of the OSNs engages the LN2 circuit to a minimal level, setting a threshold below which any sensory input is suppressed. Upon presentation of very low ethyl butyrate concentrations, the activity mediated by the high-sensitivity *Or42b *is filtered out. For intermediate odorant concentrations, the level of activity of *Or42b *and *Or42a *is sufficient to overcome the inhibitory feedback and robust odor responses are evoked. As the stimulus concentration increases, stronger inhibitory feedback ensures that OSN activity level remains within the dynamic range of the *Or42a *and *Or42b *PNs. At very high concentrations, inhibitory feedback is further strengthened by the recruitment of low-sensitivity receptors, such as *Or35a*. We propose that reducing the number of functional OSNs is likely to impair the LN2 circuit gain-control mechanism. We found that the activity of a single functional OSN was insufficient to activate LN2 neurons at any concentration tested. The activity elicited within a single functional OSN is, therefore, directly transmitted to its cognate PN. When two or more OSNs are active, sufficient activity exists to recruit the LN2 circuit. For low to moderately high concentrations, the stimulus intensity is within the dynamic range of the OSN and chemotaxis is observed. At higher concentrations, the unfiltered activity saturates such that as the OSN reaches the limit of its dynamic range, changes in odor concentrations cannot be encoded and an avoidance response is triggered. The avoidance behavior to high concentration may be triggered by saturation of PN responses or by a mechanism outside the antennal lobe involving higher brain centers.

These results contrast with recent investigation of the adult antennal lobe by Olsen and Wilson [20] and Root *et al*. [21]. Both of these groups found clear evidence of pre-synaptic inhibition, whereby inhibitory LNs feed back and suppress the firing of OSNs. We found no evidence of such presynaptic inhibition in the larva because OSNs have the same response properties in wild-type and single-OSN-functional animals. If presynaptic mechanisms of gain control operated in the larva, we would expect higher activity in OSN terminals in the single-OSN-functional animals.

A definitive genetic test of the hypothesis that LN2 neurons modulate sensitivity of PNs to ethyl butyrate at high concentrations would be to inactivate or silence these neurons in a wild-type larva, with the prediction that such animals should be more sensitive to low concentrations of ethyl butyrate and should begin avoiding high concentrations of this ester. Unfortunately, the LN2-Gal4 line is expressed in additional neurons in the mushroom body calyx and ventral ganglion. Larvae in which we have expressed the cell-autonomous toxin diphtheria toxin [45], or an inhibitor of evoked synaptic release, tetanus toxin [46], were either dead or sluggish, respectively, precluding meaningful behavioral analysis. Future work to identify more selective genetic reagents that enable us to manipulate these neurons will permit a critical test of this hypothesis.

## Conclusion

Sensory systems are adapted to the evolutionary and ethological needs of individual animals. Recognition of bitter tastes that signal potential poisons occurs at much lower concentrations than detection of sweet taste, which has evolved to evaluate food sources rich in carbohydrates and is thus most activated by high sugar concentrations [47,48]. Similarly, detection of alarm and sex pheromones by the olfactory system of insects is optimized for high sensitivity and selectivity [49-51].

What would be the advantage for larvae to ignore low concentrations of odors and retain strong and consistent attraction to high odor concentrations? Embryos are deposited directly onto food by female flies, who choose optimal sites of oviposition based on both the quality of available food and on preexisting egg populations [52,53]. Field studies of *Drosophila *species have documented that these insects feed on yeast growing on rotting fruit or plant parts [54], and that some species strongly prefer one yeast species over others [55]. As larvae hatch directly on their food source, it is essential that they can tolerate high odor concentrations and remain attracted to them without being distracted by low-concentration stimuli [56]. A similar neural mechanism, with a similar adaptive function for finding even concentrated food odors attractive, is likely to be adaptive for all higher animals. Beyond this, our data provide a plausible model for concentration-invariant olfactory perception observed in human psychophysical experiments.

## Materials and methods

### *Drosophila *strains

Larvae (*D. melanogaster*) were raised on standard medium at 18°C. Genotypes and sources of strains used in this work are: UAS-G-CaMP1.3 on the X chromosome [10] and UAS-G-CaMP1.3 on III chromosome (from A Wong and R Axel); UAS-G-CaMP1.6 [57] (from J Nakai via A Fiala); OR-Gal4 lines [22,58]; *Or83b*^1^, *Or83b*^2^, UAS-*Or83b *[35]; LN1-Gal4 and LN2-Gal4 [40]; GH146 [59] and GH298 [59] (from R Stocker); *Or42a*-nsyb:tdTomato (described below); Krasavietz-Gal4 [39] (from J Dubnau). All genotypes and strains used in this paper are listed in Additional data file 1.

Only female larvae were used for imaging. Thus, flies for OSN imaging carried eight independent insertions of UAS-G-CaMP1.3. For LN and PN imaging, we used a newer version of G-CaMP (1.6) that is about 40 times brighter and more photostable than G-CaMP1.3 [57], because G-CaMP1.3 provided insufficient signal-to-noise resolution for LN and PN imaging. pUAST-G-CaMP1.6 [57] was provided by A Fiala and used to generate transgenic strains by standard methods. Two copies of UAS-G-CaMP1.6 on the X chromosome were sufficient to image LNs and PNs.

*Or35a*-*Or83b*, *Or42a*-*Or83b *and *Or42b*-*Or83b *were constructed by first subcloning the *Or83b *cDNA coding sequence into pCasPeR-AUG-Gal4-X [60], and subsequently inserting the promoter of *Or35a*, *Or42a *or *Or42b *[22,58] upstream of the *Or83b *coding sequence. These insertions were used to create *Or35a*, *Or42a*, *Or42b *and *Or42a*+*Or42b *OSN functional larvae for PN and LN2 imaging. *Or42a*-nsyb:tdTomato was constructed by first fusing the first 549 base pairs of *Drosophila *n-synaptobrevin coding sequence [61] and the entire tdTomato coding sequence derived from pRSETB-tdTomato [62] (from R Tsien) and subcloning the fused sequence into pCasPeR-AUG-GAL4-X [60], such that the *Or42a *promoter [58] was inserted upstream of the nsyb:tdTomato coding sequence.

OR-Gal4 lines inserted on the second chromosome [22,58] were used to express G-CaMP in specific OSNs. As described elsewhere [22], larvae with a single or a pair of functional OSNs were engineered by restoring the expression of *Or83b *with *OrX*-Gal4 and UAS-*Or83b *transgenes in an *Or83b*-null background [35].

### Calcium imaging

Calcium imaging was performed with an Eclipse E600FN microscope (Nikon Instruments) with a 60× water immersion lens using software (TILL VisION; TILL Photonics, Inc.) and instrumentation previously described [40]. Adult hemolymph-like (AHL) saline [10] was used for all imaging experiments. Female feeding third instar larvae were rinsed in 1× PBS and transferred to chilled AHL saline for dissection. The larval head was removed, and fat body, salivary gland, and the digestive system posterior to the proven-triculus were removed. The preparation was inserted into a hole punched through a western blot vinyl membrane glued to a 24 mm × 20 mm plastic cover slip (HybriSlip, Grace Bio-Labs), with the head facing down and the brain facing up. Low melting agarose (1.5%; Type IX-A, Sigma-Aldrich) in AHL was applied to the brain side of the preparation and the sample was chilled for 3 minutes at 4°C. Samples were then transferred to the imaging microscope, and saline was applied on top of the agarose layer. Although peristaltic motion of the head and stable odor-evoked responses in each sample were typically obtained for up to 3 h, each sample preparation was imaged for only 1 h.

Odors were obtained from Sigma-Aldrich or Fluka at high purity and were diluted in paraffin oil. Odor concentrations for imaging are indicated as dilutions of odor in paraffin oil (v:v, hence (Volume of odor)/(Volume of paraffin oil)). For example, 10^-2 ^dilution indicates that one volume of an odor is diluted with 100 volumes of paraffin oil. Fresh dilutions were prepared monthly. Common names and Chemical Abstracts Service (CAS) numbers are: geranyl acetate (105-87-3), ethyl acetate (141-78-6), ethyl butyrate (105-54-4), isoamyl acetate (123-92-2), pentyl acetate (628-63-7), hexyl acetate (142-92-7), octyl acetate (112-14-1), 1-Hexanol (111-27-3), 1-Octen-3-ol (3391-86-4), isoamyl alcohol (123-51-3), cyclohexanol (108-93-0), 2-Phenyl ethanol (60-12-8), 2-Heptanone (110-43-0), cyclohexanone (108-94-1), E2-Hexenal (6728-26-3), octanal (124-13-0), acetophenone (98-86-2), anisole (100-66-3), methyl salicylate (119-36-8), 4-Methyl phenol (106-44-5), acetyl furan (1192-62-7), and propyl sulfide (111-47-7).

Ten microliters of diluted odor solution was applied to a 0.25-inch filter paper (Whatman) inside a 1 ml plastic syringe (Becton-Dickinson) attached to Nalgene 890 PTFE FEP tubing (1/8 inch; Fisher Scientific) connected to a switching solenoid valve (The Lee Co.). The valve was controlled by a BPS-4 valve control box (ALA Scientific Instruments) via computer and alternated between clean air flow and the odor syringe. The tip of the odor syringe was positioned about 1 cm away from the sample. To avoid contamination, the tubing directly connecting an odor syringe was replaced after each use, an odor syringe was not used more than three times, and air around the samples was continually removed by ventilation. Charcoal-filtered and humidified air was adjusted to a flow rate of 1000 ml/minute with a flowmeter (Gilmont Instruments).

Each odor, at intervals of approximately 100 s, was applied only once unless the sample moved out of the square region of interest (typically 9 × 9 pixels) during the experiment, according to the following protocol for OSNs: 3 s pre-stimulus, 1 s odor stimulus, and 8 s post-stimulus. For PNs and LNs, the protocol was 6 s pre-stimulus, 1 s odor stimulus, and 8 s post-stimulus. The order of the odors to be tested was randomly determined for each sample and saline was replaced every 15 minutes. Images were acquired at five frames per second at an exposure time of 50 ms and a resolution of 72 × 72 pixels (binned 8 × 8) for OSNs and 96 × 96 pixels (binned 8 × 8) for PNs and LNs. Samples were excluded from analysis if responses to reference odors inserted during and at the end of each imaging experiment showed deterioration in response magnitude or onset.

Calcium-imaging data were analyzed by a custom program in IDL (ITT Visual Information Solutions, written and provided by CG Galizia and M Ditzen). Samples that showed excessive movement were discarded, and the rest underwent movement correction if necessary by shifting each frame so that a region of interest was situated on the same coordinate throughout the imaging experiment. The fluorescence value was then calculated by averaging the fluorescence intensity within the region of interest for each OSN in each frame (designated as F_n _for the nth frame). The relative change in fluorescence, or Δ*F*/*F*, for an OSN was then calculated as follows:

$${\left(\frac{\Delta F}{F}\right)}_{n}=\left(F_{n}-\frac{\sum_{i=10}^{14}F_{i}}{5}\right)/\frac{\sum_{i=10}^{14}F_{i}}{5}$$

For PNs and LNs, Δ*F*/*F *was calculated as follows:

$${\left(\frac{\Delta F}{F}\right)}_{n}=\left(F_{n}-\frac{\sum_{i=25}^{29}F_{i}}{5}\right)/\frac{\sum_{i=25}^{29}F_{i}}{5}$$

In both cases, (Δ*F*/*F*)_*n *_is thus defined as fluorescence intensity relative to the average fluorescence intensity during 1 s immediately before the onset of odor stimulation.

The first 1 s of OSN imaging and the first 4 s of PN and LN imaging were excluded from the false color-coded plots as bleaching of fluorescence was significant. No correction was made for bleaching thereafter, as odor-evoked responses were strong despite bleaching. We noticed a consistent mechanical artifact in imaging PNs in *Or35a*-functional animals, which we believe is due to the sensitivity of these cells to mechanical stimulation by changes in air flow. The time courses of Δ*F*/*F *were converted to false color-coded plots using Matlab (The Mathworks).

Response delays in the imaging data were not corrected. The only criterion we applied to an imaged sample is that the onset of response to a reference odor (for example, ethyl butyrate for the *Or42a *OSN) must fall within 200 ms after odor application. We discovered empirically that samples showing delayed responses often became unresponsive to odors after 10–15 minutes rather than the 1–3 h timeframe found for good samples. Accordingly, samples showing greater than 200 ms latency in response to reference odor were discarded from further experiments. Subtle differences in odor onset can be seen in our data set (Additional data file 1 (Figure S1: 4-Methyl phenol stimulation of *Or1a*, *Or45b*, and *Or83a*)), but given the low temporal resolution of calcium imaging, we have not emphasized these possible latency differences in our paper.

The half-maximal effective concentrations for ethyl butyrate to activate a given OSN (EC_50 _values) were calculated from calcium-imaging data in wild-type (Figure 2b) and single-OSN-functional animals (Additional data file 1 (Figure S2)). Response values at a given odor concentration were obtained by integrating the Δ*F*/*F *value for 1 s after odor onset and EC_50 _values were calculated from these data using Prism (GraphPad Software) to fit the data to the Hill equation. EC_50 _values (95% confidence interval) are as follows:

*Or35a *wild-type: 1.1 × 10^-2^-2.2 × 10^-3^

*Or35a*-functional: 1.2 × 10^-2^-3.9 × 10^-3^

*Or42a *wild-type: 1.3 × 10^-3^-6.3 × 10^-4^

*Or42a*-functional: 8.4 × 10^-4^-3.1 × 10^-4^

*Or42b *wild-type: 7.9 × 10^-5^-2.6 × 10^-5^

*Or42b*-functional: 1.3 × 10^-4^-3.9 × 10^-5^

Because the 95% confidence intervals overlap, the sensitivity to ethyl butyrate does not differ statistically between wild-type and *OrX*-functional OSNs (*p *> 0.05).

The same EC_50 _calculations were carried out for imaging at PN terminals with the exception that response values at a given odor concentration were obtained by integrating the Δ*F*/*F *value for 1 s. PN response durations are much shorter than those for OSNs (compare Figures 2b and 5e). EC_50 _values, displayed as 95% confidence intervals, are as follows:

*Or35a *5.3 × 10^-3^-2.6 × 10^-2^

*Or42a *1.4 × 10^-4^-4.2 × 10^-4^

*Or42b *(subdomain 1) 5.1 × 10^-5^-1.7 × 10^-4^

*Or42b *(subdomain 2) 4.6 × 10^-5^-2.6 × 10^-4^

### Larval behavior

Single and multiple odor source devices (Figures 3a and 4a) were constructed as previously described [26]. The concentration of ethyl butyrate was measured in gas phase by integrating infrared (IR) light absorbance along sections of the arena at a rate of one per minute and at wave number 1,758 cm^-1^. Absolute odor concentration was calculated from the Beer-Lambert law. The molar extinction coefficient of ethyl butyrate was determined in gas phase with a standard gas-flow cell: ε^ethyl butyrate ^= 315 M^-1 ^cm^-1^.

Odor dilutions were prepared in paraffin oil using a digital scale to measure the amounts of solvent and odor mixed in each dilution [29]. Using IR spectroscopy, the concentration of a representative subset of odor dilutions was systematically controlled in liquid phase (data not shown). All odor sources had a volume of 10 μl.

Odor-evoked behavior of single larvae was monitored and quantified as previously described [26]. Approximately 30 s after odor source loading, a single larva was introduced under the source (single odor source assay) or at the low concentration end of the odorant line (multiple odor source assay). For the single odor source assay, recordings lasted 5 minutes unless the animal contacted any walls of the arena. Fifteen individuals were tested for each genotype and source concentration (Figure 3), and each animal was tested in a fresh arena. For the multiple odor source assay, recordings lasted a maximum of 3 minutes and were stopped as soon as the animals reached the highest odor concentration. Twenty to thirty individuals were tested for each genotype and gradient amplitude (Figure 4), and each arena was used to test five consecutive animals before being replaced.

For the single odor source assay, spatial dispersion of paths was quantified relative to the odor source, which produced a radially symmetric odor distribution (Figure 3b). The arena was partitioned into concentric 0.25-cm rings (distance bins) centered on the source position. Positions falling out of the largest ring contained in the arena are not reported in Figure 3. The fraction of positions comprising each distance bin was computed for every path. Medians were then calculated over the relative occupancy distributions of 15 larvae. For a given genotype and source concentration, medians associated with each distance bin were compared to the *Or83b*^-/- ^control using a Wilcoxon rank-sum test, adjusted by a Bonferroni correction to maintain the confidence level at 5%.

For the multiple odor source assay, binary dilutions of source concentrations were used to generate gradients with an exponential profile along their length (Figure 4a–c). The surface of the arena was partitioned into three zones (Z1, Z2, and Z3) on the basis of the topography of the gradient displayed in Figure 4c. The fraction of positions comprising each zone was computed for individual tracks, and distributions of *n* = 30 larvae were calculated and are presented as boxplots (Figure 4d). The alignment of individual paths with the odor gradient was quantified by a previously described combined chemotaxis score [26], ranging between 0 (disregard for odorant line) and 1 (perfect alignment with odorant line).

### Immunostaining

Whole-mount larval brain immunostaining was carried out as previously described [22] with the following antibodies: mouse anti-*Drosophila *choline acetyltransferase (ChAT4B1, 1:100; this monoclonal antibody developed by PM Salvaterra was obtained from the Developmental Studies Hybridoma Bank developed under the auspices of the NICHD and maintained by Department of Biological Sciences, University of Iowa, Iowa City, IA 52242, USA); rabbit anti-GFP (Molecular Probes, 1:1000); mouse anti-GABA (Sigma, 1:1000); mouse nc82 (gift from R Stocker, 1:10); goat anti-rabbit Alexa 488 (Molecular Probes, 1:100); goat anti-mouse Cy3 (Jackson ImmunoResearch, 1:100). Images were acquired with a Zeiss LSM510 confocal microscope.

## Additional data files

Additional data file 1 contains additional Figures S1–S6 and information on genotypes of all *Drosophila *strains used in this paper.

## Authors' contributions

KA carried out the imaging and immunostaining in Figures 1, 2, 5, 6, 7 and S1–S5. ML supervised and SP carried out the behavioral experiments in Figures 3 and 4. Analysis of the behavioral data was jointly performed by ML and SP. LBV directed the project and together with the other authors wrote the paper.

## Supplementary Material

Additional file 1 Additional Figures S1–S6 and information on genotypes of all *Drosophila *strains used in this paper. Click here for file
