# Supplementary material for: A circuit supporting concentration-invariant odor perception in Drosophila
Source: J Biol. 2009 Jan 26;8(1):9. doi: 10.1186/jbiol108 (PMC2656214; doi:10.1186/jbiol108)
Supplement: Additional file 1 — Additional Figures S1–S6 and information on genotypes of all Drosophila strains used in this paper. [file jbiol108-S1.pdf]

Supplementary Figures

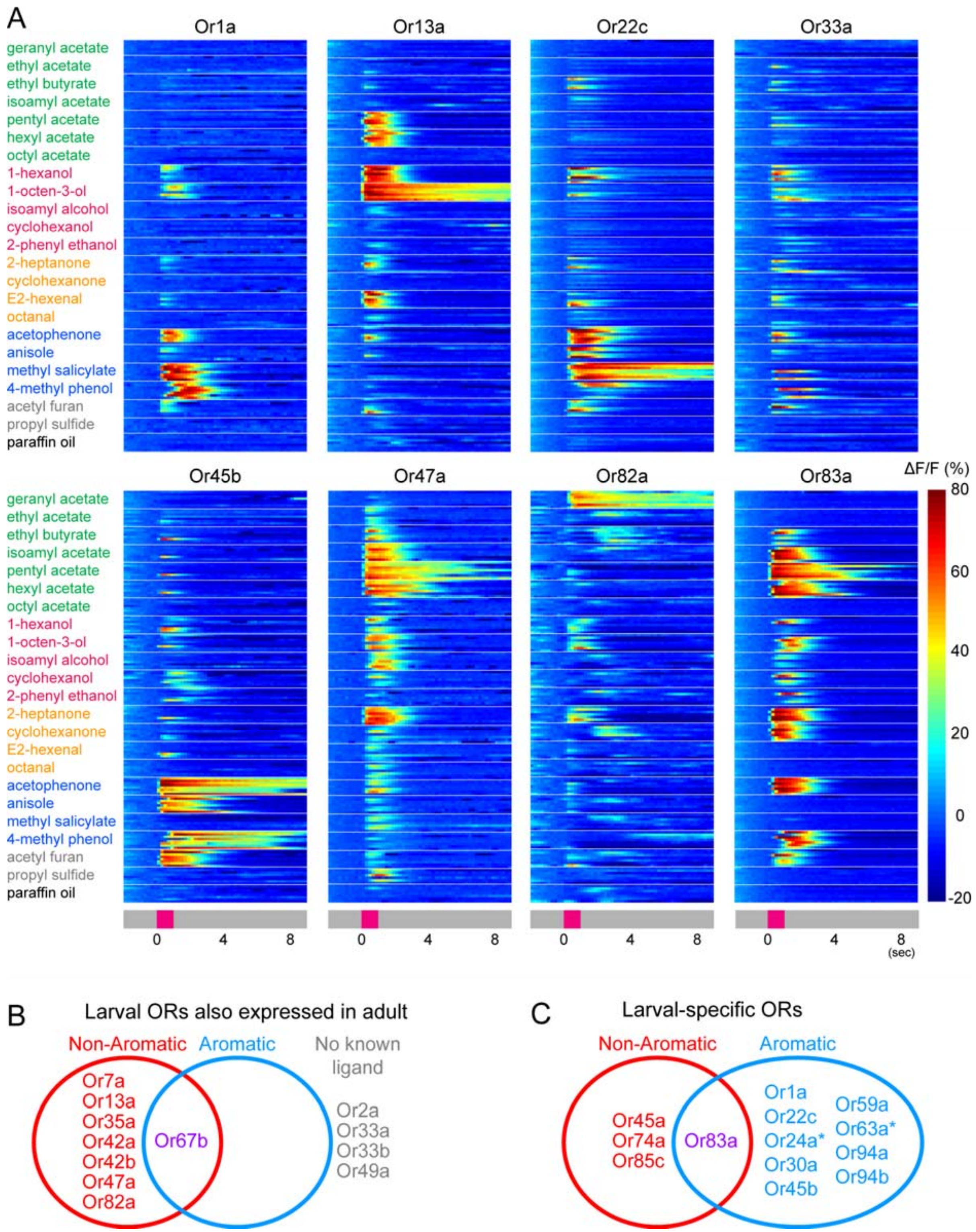

Figure S1. Ligand tuning of larval olfactory neurons

(A) Odor response profiles of 8 OSNs, measured at axon termini of a given OSN in the antennal lobe, against a panel of 22 odorants (10^{-2} dilution) and paraffin oil (solvent), are shown as described in Figure 1d. Traces from n=7-9 animals per stimulus are stacked. Two OSNs were routinely labeled with G-CaMP by crossing two homozygous strains carrying each of the 11 Gal4 drivers (including *Or35a*, *Or42a*, and *Or42b*) together with UAS-G-CaMP, allowing us to image simultaneously from two OSNs. Combinations were chosen to maximize separation of the two axon terminals of the labeled OSNs (Figure 1 b-c: see "Genotype of *Drosophila* strains used in this paper" for the complete list of genotypes used).

(B) Summary of ligand specificity of ORs expressed in both larval and adult OSNs, from this study and from the literature [1]. No ligands have been found to activate the four receptors in gray (*Or2a*, *Or33b* [1]; *Or33a*, this study; *Or49a*, [2, 3]).

(C) Summary of ligand specificity of larval-specific ORs from this study and previous studies [3, 4]. Calcium imaging carried out in this study showed that larval OSNs can be categorized into aromatic odor-sensitive (*Or1a*, *Or22c*, *Or24a**: preliminary data, *Or45b*) and non-aromatic odor-sensitive (*Or13a*, *Or35a*, *Or42a*, *Or42b*, *Or47a*, *Or82a*) classes, consistent with previous findings [3,4]. Preliminary imaging data from this study show that *Or63a* is activated by aromatic odors (indicated by * on the figure).

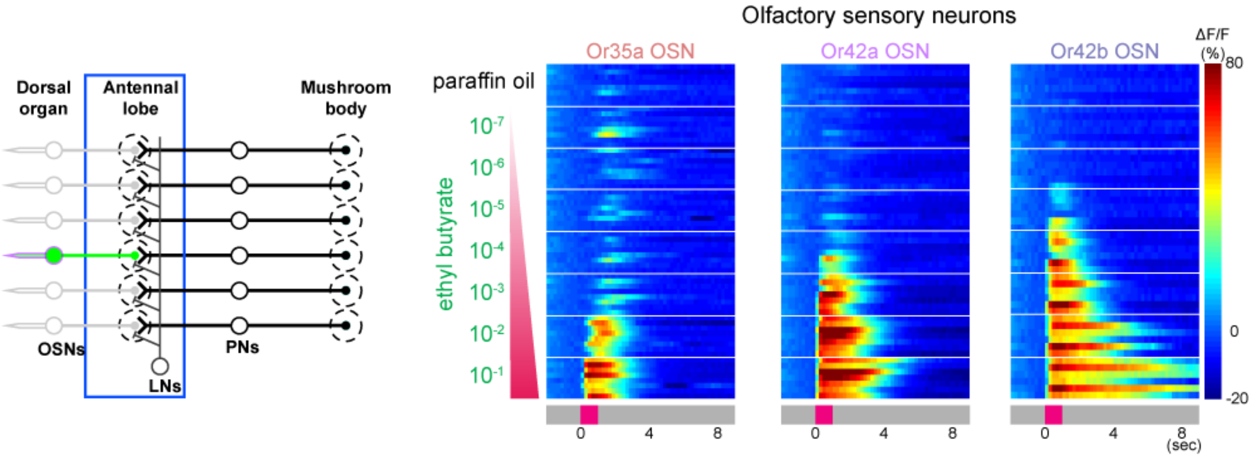

Figure S2. Concentration-dependent responses to ethyl butyrate in olfactory neurons in single OSN functional animals

Responses of *Or35a*, *Or42a*, and *Or42b* OSNs in single-functional *Or83b* mutant larvae to an ethyl butyrate concentration series and paraffin oil (solvent) represented as % ΔF/F (scale at right). Traces from n=6-8 animals per genotype and stimulus are stacked.

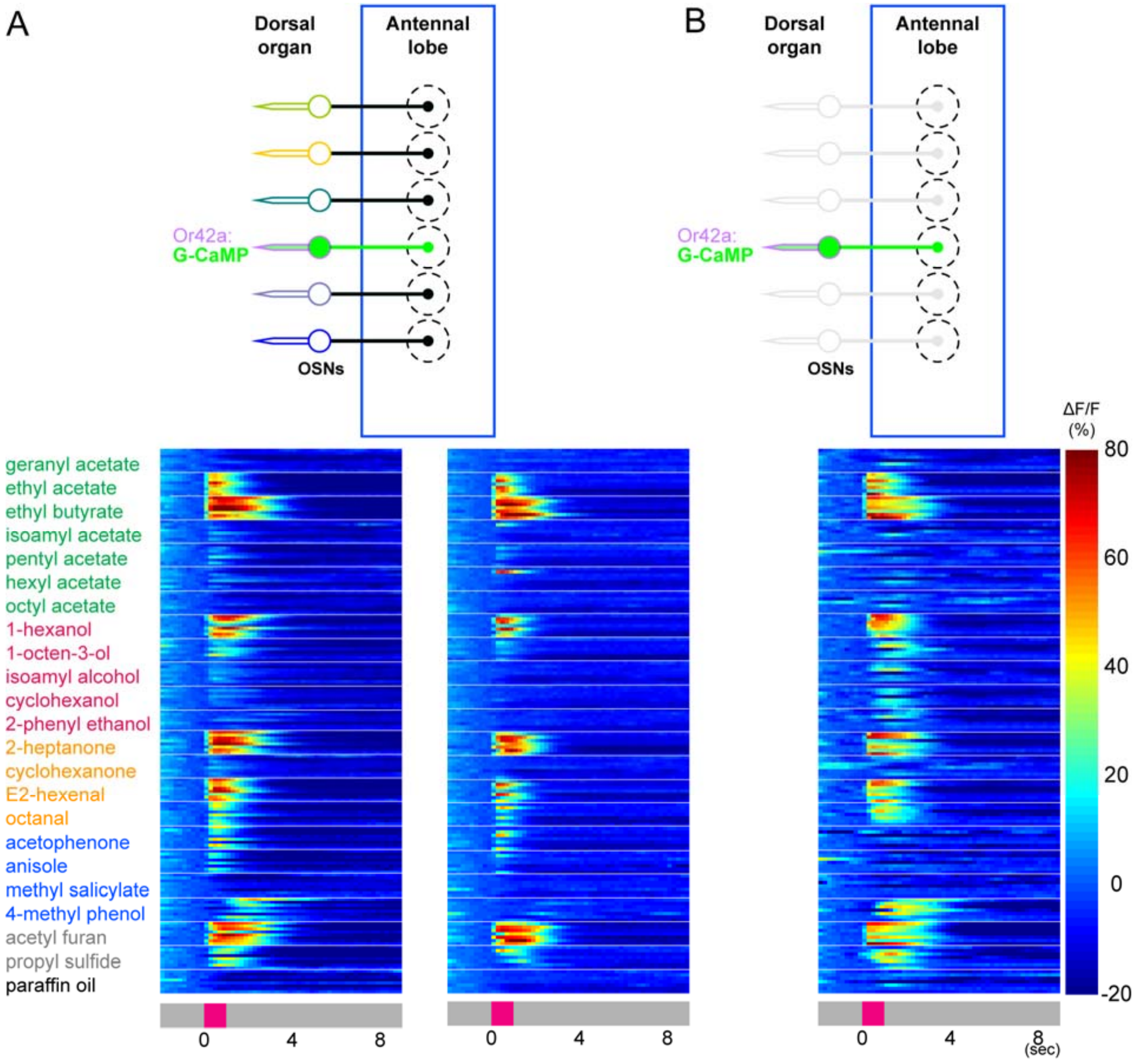

Figure S3. The odor response profile of a larval OSN is stable

(A-B) Schematic for measuring functional activation of larval OSNs in the antennal lobe (blue box) of wild type (A) and *Or42a*-functional (B) larvae. Two independent insertions of *Or42a*-Gal4 (Gal4#1 and Gal4#2) were used for the imaging experiments: (A) wild type: Gal4#1, left; Gal4#2, right. (B) *Or42a*-functional: Gal4#2. Data are represented as false color-coded time traces (% ΔF/F; scale at bottom right). Traces from n=7 animals per stimulus are stacked. The left panel in (A) is reproduced from Figure 2a.

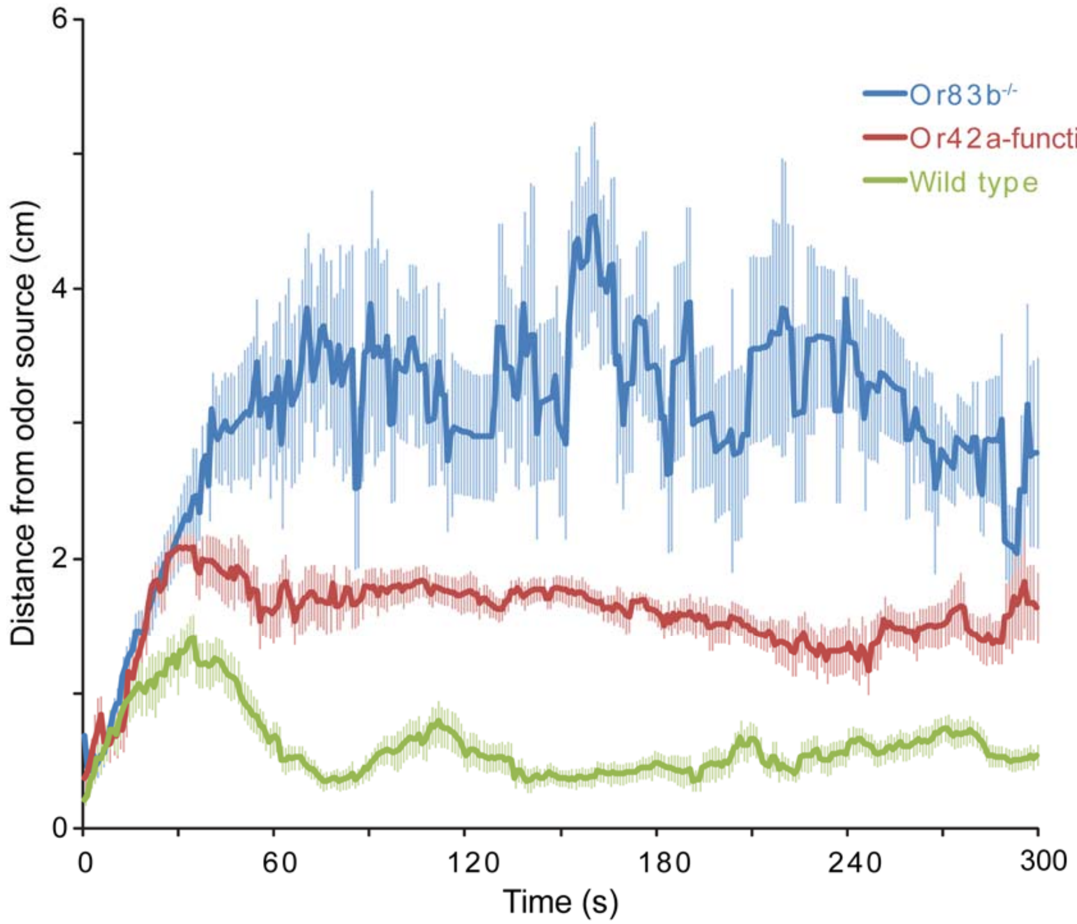

Figure S4. Time-dependent odor-evoked behavior in the single odor source assay
Time course of the mean distance to the odor source calculated from a pool of 15 trajectories for three genotypes of larvae. The mean distances to the odor source calculated over consecutive 1 second bins are indicated by the dark line and the standard deviations of the means (error bars) are indicated by the shaded area surrounding the dark line. *Or42a*-functional (red) and wild type (green) larvae were tested with a source concentration of 15mM ethyl butyrate while *Or83b* mutant (blue) larvae were tested with 60mM ethyl butyrate. Animals are introduced under the odor source shortly before time t=0 seconds (s).

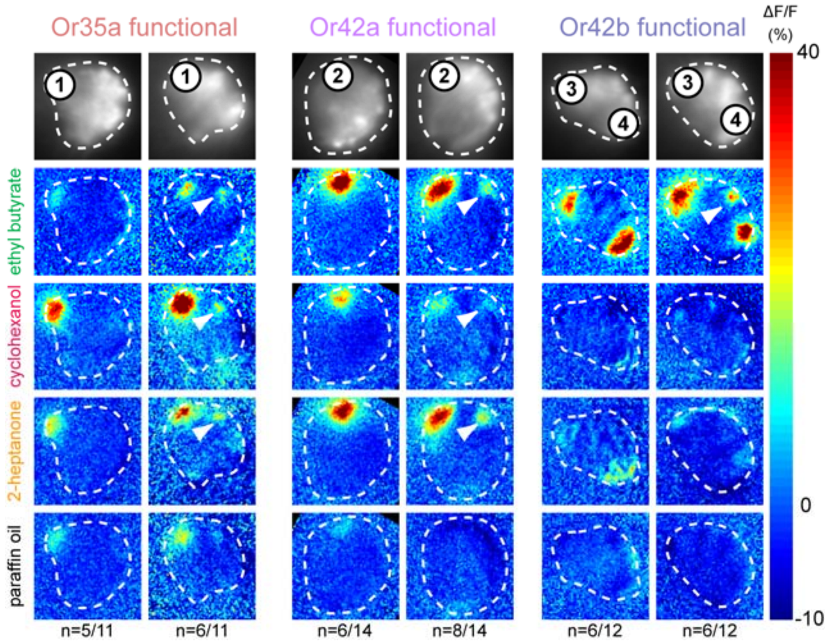

Figure S5. Secondary odor-evoked signals in the mushroom body

PN imaging data from *Or35a*-, *Or42a*-, or *Or42b*-functional animals showing representative images from two types of samples that differ in their degree of activation of secondary glomeruli. Data from Figure 5c are reproduced in each of the left columns. The right columns show imaging samples with weaker activation of secondary glomeruli (white arrowhead). The top row shows intrinsic mushroom body G-CaMP fluorescence and bottom four rows show false color-coded image of mushroom body taken 600 millisec after stimulus onset, and represented as ΔF/F (%) (scale at the right). The number of samples with or without secondary glomeruli is shown at the bottom of each column.

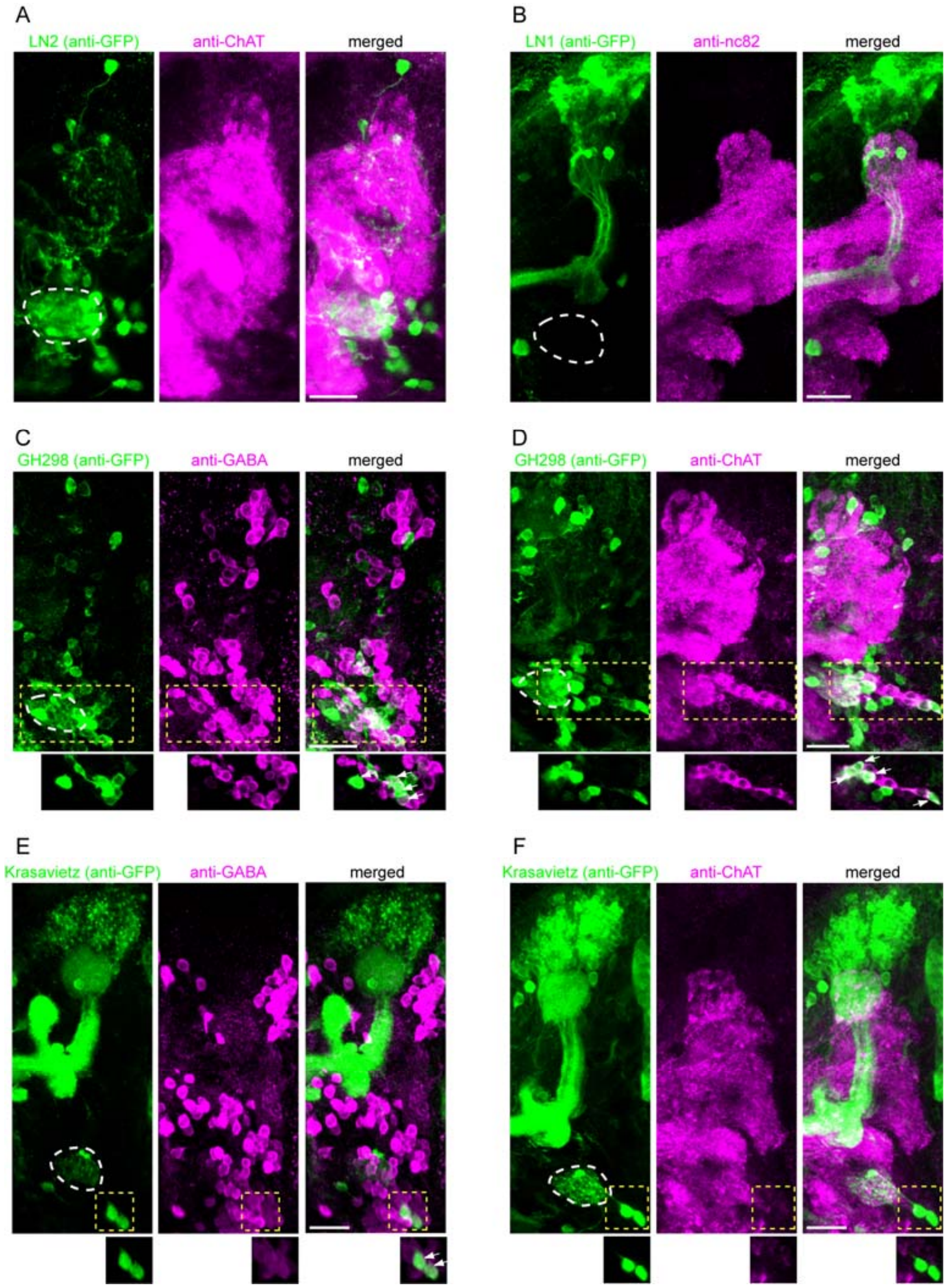
A
LN2 (anti-GFP)
anti-ChAT
merged
B
LN1 (anti-GFP)
anti-nc82
merged
C
GH298 (anti-GFP)
anti-GABA
merged
D
GH298 (anti-GFP)
anti-ChAT
merged
E
Krasavietz (anti-GFP)
anti-GABA
merged
F
Krasavietz (anti-GFP)
anti-ChAT
merged

Figure S6: Characterization of potential local interneuron-selective Gal4 lines in the larval brain

(A) Whole mount immunofluorescence staining of a *LN2-Gal4; UAS-G-CaMP* larva. Green (left) indicates anti-GFP staining and magenta (middle) indicates *Drosophila* choline acetyltransferase (ChAT) staining. For this and all subsequent panels, the right image is a merged image of the green and magenta channels.

(B) Whole mount immunofluorescence staining of a *LN1-Gal4; UAS-GFP* larva. Green (left) indicates anti-GFP staining and magenta (middle) indicates nc82 staining, which labels neuropil.

(C) Whole mount immunofluorescence staining of a *GH298; UAS-G-CaMP* larva. Green (left) indicates anti-GFP staining and magenta (middle) indicates GABA staining.

(D) Whole mount immunofluorescence staining of a *GH298; UAS-G-CaMP* larva. Green (left) indicates anti-GFP staining and magenta (middle) indicates ChAT staining.

(E) Whole mount immunofluorescence staining of a *Krasavietz-Gal4; UAS-G-CaMP* larva. Green (left) indicates anti-GFP staining and magenta (middle) indicates GABA staining.

(F) Whole mount immunofluorescence staining of a *Krasavietz-Gal4; UAS-G-CaMP* larva. Green (left) indicates anti-GFP staining and magenta (middle) indicates ChAT staining.

All images are stacked confocal images that cover the left hemisphere of the larval brain neuropil including the larval antennal lobe (indicated with white dotted line in anti-GFP staining image) and the mushroom body. Scale bar = 20μm. In (C-F), cell bodies from 1-3 optical sections from rectangular area shown in dotted yellow line are shown at the bottom. Co-localization of anti-GFP staining and either GABA or choline acetyltransferase is indicated by a white arrow in the merged image.

Genotypes of *Drosophila* strains used in this paper

Figures in main manuscript:

<u>Figure 1</u>

Or35a, *Or42a* — imaged from *Or35a* and *Or42a* OSNs in animals of these two genotypes:

UAS-G-CaMP1.3*/UAS-G-CaMP1.3*; *Or35a*-Gal4/*Or42a*-Gal4#1; UAS-G-CaMP1.3*/UAS-G-CaMP1.3*

<u>Figure 2</u>

Or35a — imaged from *Or35a* OSNs in animals of these two genotypes:

UAS-G-CaMP1.3*/UAS-G-CaMP1.3*; *Or22c*-Gal4/*Or35a*-Gal4; UAS-G-CaMP1.3*/UAS-G-CaMP1.3*

UAS-G-CaMP1.3*/UAS-G-CaMP1.3*; *Or35a*-Gal4/*Or42a*-Gal4#1; UAS-G-CaMP1.3*/UAS-G-CaMP1.3*

Or42a — imaged from *Or42a* OSNs in animals of this genotype:

UAS-G-CaMP1.3*/UAS-G-CaMP1.3*; *Or35a*-Gal4/*Or42a*-Gal4#1; UAS-G-CaMP1.3*/UAS-G-CaMP1.3*

Or42b — imaged from *Or42b* OSNs in animals of these two genotypes:

UAS-G-CaMP1.3*/UAS-G-CaMP1.3*; *Or22c*-Gal4/*Or42b*-Gal4; UAS-G-CaMP1.3*/UAS-G-CaMP1.3*

UAS-G-CaMP1.3*/UAS-G-CaMP1.3*; *Or42b*-Gal4/*Or45b*-Gal4; UAS-G-CaMP1.3*/UAS-G-CaMP1.3*

<u>Figure 3 and Figure 4</u>

Wild type — w^{1118}

Or83b$^{-/-}$ — *w/w*; +/+; UAS-*Or83b*, *Or83b*^{1}/*Or83b*^{2}

Or35a-functional — *w/w*; *Or35a*-Gal4/*Or35a*-Gal4; UAS-*Or83b*, *Or83b*^{1}/*Or83b*^{2}

Or42a-functional — *w/w*; *Or42a*-Gal4#1/*Or42a*-Gal4#1; UAS-*Or83b*, *Or83b*^{1}/*Or83b*^{2}

Or42b-functional — *w/w*; *Or42b*-Gal4/*Or42b*-Gal4; UAS-*Or83b*, *Or83b*^{1}/*Or83b*^{2}

Or42a+Or42b-functional	*w/w*; *Or42a*-Gal4#1/*Or42b*-Gal4; UAS-*Or83b*, *Or83b*^{1}/*Or83b*^{2}

Figure 5

Or35a-functional	UAS-G-CaMP1.6/UAS-G-CaMP1.6; GH146/CyO; *Or35a-Or83b, Or83b*^{1}/*Or83b*^{1}
Or42a-functional	UAS-G-CaMP1.6/UAS-G-CaMP1.6; GH146/CyO; *Or42a-Or83b, Or83b*^{1}/*Or83b*^{1}
Or42b-functional	UAS-G-CaMP1.6/UAS-G-CaMP1.6; GH146/CyO; *Or42b-Or83b, Or83b*^{1}/*Or83b*^{1}

Figure 6

Wild type	UAS-G-CaMP1.6, LN2-Gal4/ UAS-G-CaMP1.6, LN2-Gal4; Or42a-nsyb:tdTomato/+; +/+
Or42a-functional	UAS-G-CaMP1.6, LN2-Gal4/ UAS-G-CaMP1.6, LN2-Gal4; Or42a-nsyb:tdTomato/+; *Or42a-Or83b, Or83b*^{1}/*Or83b*^{1}
Or42b-functional	UAS-G-CaMP1.6, LN2-Gal4/ UAS-G-CaMP1.6, LN2-Gal4; Or42a-nsyb:tdTomato/+; *Or42b-Or83b, Or83b*^{1}/*Or83b*^{1}
Or42a+Or42b-functional	UAS-G-CaMP1.6, LN2-Gal4/ UAS-G-CaMP1.6, LN2-Gal4; Or42a-nsyb:tdTomato/+; *Or42a-Or83b, Or83b*^{1}/*Or42b-Or83b, Or83b*^{1}

Figure 7

Or42a-functional	UAS-G-CaMP1.6/UAS-G-CaMP1.6; GH146/CyO; *Or42a-Or83b, Or83b*^{1}/*Or83b*^{1}
Or42b-functional	UAS-G-CaMP1.6/UAS-G-CaMP1.6; GH146/CyO; *Or42b-Or83b, Or83b*^{1}/*Or83b*^{1}
Or42a+Or42b-functional	UAS-G-CaMP1.6/UAS-G-CaMP1.6; GH146/CyO; *Or42a-Or83b, Or83b*^{1}/*Or42b-Or83b*, *Or83b*^{1}

Figures in Supplementary Materials:

Figure S1

Or1a imaged from *Or1a* OSNs in animals of this genotype:

UAS-G-CaMP1.3*/UAS-G-CaMP1.3*; *Or1a*-Gal4/*Or47a*-Gal4; UAS-G-CaMP1.3*/UAS-G-CaMP1.3*

Or13a imaged from *Or13a* OSNs in animals of this genotype:

UAS-G-CaMP1.3*/UAS-G-CaMP1.3*; *Or13a*-Gal4/*Or33a*-Gal4; UAS-G-CaMP1.3*/UAS-G-CaMP1.3*

Or22c imaged from *Or22c* OSNs in animals of these two genotypes:

UAS-G-CaMP1.3*/UAS-G-CaMP1.3*; *Or22c*-Gal4/*Or35a*-Gal4; UAS-G-CaMP1.3*/UAS-G-CaMP1.3*

UAS-G-CaMP1.3*/UAS-G-CaMP1.3*; *Or22c*-Gal4/*Or42b*-Gal4; UAS-G-CaMP1.3*/UAS-G-CaMP1.3*

Or33a imaged from *Or33a* OSNs in animals of this genotype:

UAS-G-CaMP1.3*/UAS-G-CaMP1.3*; *Or13a*-Gal4/*Or33a*-Gal4; UAS-G-CaMP1.3*/UAS-G-CaMP1.3*

Or45b imaged from *Or45b* OSNs in animals of this genotype:

UAS-G-CaMP1.3*/UAS-G-CaMP1.3*; *Or42b*-Gal4/*Or45b*-Gal4; UAS-G-CaMP1.3*/UAS-G-CaMP1.3*

Or47a imaged from *Or47a* OSNs in animals of this genotype:

UAS-G-CaMP1.3*/UAS-G-CaMP1.3*; *Or1a*-Gal4/*Or47a*-Gal4; UAS-G-CaMP1.3*/UAS-G-CaMP1.3*

Or82a imaged from *Or82a* OSNs in animals of this genotype:

UAS-G-CaMP1.3*/UAS-G-CaMP1.3*; *Or42a*-Gal4#2/*Or82a*-Gal4; UAS-G-CaMP1.3*/UAS-G-CaMP1.3*

Or83a imaged from *Or83a* OSNs in animals of this genotype:

UAS-G-CaMP1.3*/UAS-G-CaMP1.3*; *Or83a*-Gal4/CyO; UAS-G-CaMP1.3*/UAS-G-CaMP1.3*

Figure S2

Or35a-functional UAS-G-CaMP1.3*/UAS-G-CaMP1.3*; *Or35a*-Gal4; UAS-G-CaMP1.3, $Or83b^{1}$, UAS-*Or83b*/UAS-G-CaMP1.3, $Or83b^{2}$

Or42a-functional UAS-G-CaMP1.3*/UAS-G-CaMP1.3*; *Or42a*-Gal4#1; UAS-G-CaMP1.3, $Or83b^{1}$, UAS-*Or83b*/UAS-G-CaMP1.3, $Or83b^{2}$

Or42b-functional UAS-G-CaMP1.3*/UAS-G-CaMP1.3*; *Or42b*-Gal4; UAS-G-CaMP1.3, $Or83b^{1}$, UAS-*Or83b*/UAS-G-CaMP1.3, $Or83b^{2}$

Figure S3

Or42a OSN (Gal4#1) imaged from *Or42a* OSNs in animals of this genotype:

UAS-G-CaMP1.3*/UAS-G-CaMP1.3*; *Or35a*-Gal4/*Or42a*-Gal4#1; UAS-G-CaMP1.3*/UAS-G-CaMP1.3*

Or42a OSN (Gal4#2) imaged from *Or42a* OSNs in animals of this genotype:

UAS-G-CaMP1.3*/UAS-G-CaMP1.3*; *Or42a*-Gal4#2/*Or82a*-Gal4; UAS-G-CaMP1.3*/UAS-G-CaMP1.3*

Or42a-functional imaged from *Or42a* OSNs in animals of this genotype:

UAS-G-CaMP1.3*/UAS-G-CaMP1.3*; *Or42a*-Gal4#2/ *Or42a*-Gal4#2; UAS-G-CaMP1.3, $Or83b^{1}$, UAS-*Or83b*/ UAS-G-CaMP1.3, $Or83b^{1}$, UAS-*Or83b*

Figure S4

Wild type w^{1118}

$Or83b^{-/-}$ *w/w*; +/+; UAS-*Or83b*, $Or83b^{1}/Or83b^{2}$

Or42a-functional *w/w*; *Or42a*-Gal4#1/*Or42a*-Gal4#1; UAS-*Or83b*, $Or83b^{1}/Or83b^{2}$

Figure S5

Or35a-functional UAS-G-CaMP1.6/ UAS-G-CaMP1.6; GH146/CyO; *Or35a-Or83b,* $Or83b^{1}/Or83b^{1}$

Or42a-functional UAS-G-CaMP1.6/ UAS-G-CaMP1.6; GH146/CyO; *Or42a-Or83b,* $Or83b^{1}/Or83b^{1}$

Or42b-functional	UAS-G-CaMP1.6/ UAS-G-CaMP1.6; GH146/CyO; *Or42b-Or83b, Or83b*[1]*/Or83b*[1]

Figure S6

LN2	LN2-Gal4, UAS-G-CaMP1.6; +; +
LN1	LN1-Gal4; +; UAS-GFP/+
GH298	UAS-G-CaMP1.6/+;+;GH298/+
Krasavietz	UAS-G-CaMP1.6; +; Krasavietz-Gal4

*indicates two copies of this transgene inserted on a single chromosome

REFERENCES

1. Hallem EA, Carlson JR: **Coding of odors by a receptor repertoire**. *Cell* 2006, **125**(1):143-160.

2. Fishilevich E, Domingos AI, Asahina K, Naef F, Vosshall LB, Louis M: **Chemotaxis behavior mediated by single larval olfactory neurons in *Drosophila***. *Curr Biol* 2005, **15**(23):2086-2096.

3. Kreher SA, Kwon JY, Carlson JR: **The molecular basis of odor coding in the *Drosophila* larva**. *Neuron* 2005, **46**:445-456.

4. Kreher SA, Mathew D, Kim J, Carlson JR: **Translation of sensory input into behavioral output via an olfactory system**. *Neuron* 2008, **59**(1):110-124.
